# CBP and P300 regulate distinct gene networks required for human primary myoblast differentiation and muscle integrity

**DOI:** 10.1038/s41598-018-31102-4

**Published:** 2018-08-22

**Authors:** Lucas Fauquier, Karim Azzag, Marco Antonio Mendoza Parra, Aurélie Quillien, Manon Boulet, Sarah Diouf, Gilles Carnac, Lucas Waltzer, Hinrich Gronemeyer, Laurence Vandel

**Affiliations:** 1 Centre de Biologie du Développement (CBD), Centre de Biologie Intégrative (CBI), Université de Toulouse, CNRS, UPS, Toulouse, France; 2 Present Address: Lillehei Heart Institute, Department of Medicine, University of Minnesota, Minneapolis, MN USA; 3 Institut de Génétique et de Biologie Moléculaire et Cellulaire (IGBMC), CNRS UMR 7104, INSERM U964, University of Strasbourg, Illkirch, France; 4 Present Address: Universite Clermont Auvergne, CNRS, Inserm, GReD, F-63000 Clermont-Ferrand I, France; 5 Inserm U1046-UMR CNRS 9214, Physiologie et Médecine Expérimentale du cœur et des muscles PHYMEDEXP, CHU A. De Villeneuve, Bâtiment Crastes de Paulet, 371 avenue du doyen Giraud, F-34295 Montpellier cedex 5, France

## Abstract

The acetyltransferases CBP and P300 have been implicated in myogenesis in mouse immortalized cell lines but these studies focused only on the expression of a handful of myogenic factors. Hence, the respective role of these two related cofactors and their impact at global scale on gene expression rewiring during primary myoblast differentiation remain unknown. Here, we characterised the gene networks regulated by these two epigenetic enzymes during human primary myoblast differentiation (HPM). We found that CBP and p300 play a critical role in the activation of the myogenic program and mostly regulate distinct gene sets to control several aspects of HPM biology, even though they also exhibit some degree of redundancy. Moreover, CBP or P300 knockdown strongly impaired muscle cell adhesion and resulted in the activation of inflammation markers, two hallmarks of dystrophic disease. This was further validated in zebrafish where inhibition of CBP and P300 enzymatic activities led to cell adhesion defects and muscle fiber detachment. Our data highlight an unforeseen link between CBP/P300 activity and the emergence of dystrophic phenotypes. They thereby identify CBP and P300 as mediators of adult muscle integrity and suggest a new lead for intervention in muscular dystrophy.

## Introduction

Transcriptional regulation of gene expression is the primary mechanism controlling cellular processes such as cell proliferation and differentiation. It is a highly coordinated process that requires the recruitment of transcription factors (TF), chromatin modifying factors and the basal transcriptional machinery to regulatory regions of the genome. Among chromatin-modifying enzymes, the cAMP response element-binding protein (CREB) binding protein (also called CBP, CREBBP or KAT3A) and the highly related protein P300 (also known as KAT3B) harbour a histone acetyltransferase (HAT) activity and activate transcription by acetylating specific lysine residues on histones but also on non histone proteins such as TF or transcription coregulators^1,2^. Both CBP and P300 are involved in multiple signalling pathways and various biological processes as they are recruited to their target genes *via* a wide spectrum of transcription factors, more than 400 having been identified to date (for a review^3^).

Consistent with their function as transcriptional co-activators, CBP and P300 play a crucial role in development. Mice harbouring a homozygous deletion of either *P300* or *Cbp* die at early stage of embryonic development and exhibit distinct phenotypes, indicating that these two factors display different functions *in vivo*^4,5^. Interestingly though, *P300*^+/–^*/Cbp*^+/–^ double heterozygous mice die before birth, suggesting that CBP and P300 also exhibit some functional redundancy^4^. In contrast to the *in vivo* situation, most studies with tissue culture cells suggested similar functions for P300 and CBP, and their distinctive roles remain largely elusive. For instance, ChIP-sequencing analyses showed that the vast majority of the genes bound by CBP and P300 were common in cell cycle synchronized cells^6^. Of note though, another study trying to address the functional connections between genome-wide recruitment of CBP and gene expression showed that the majority of CBP recruitment events do not stimulate nearby genes^7^. Moreover, higher order chromosome structures mediated by P300 and CBP are important in maintaining embryonic stem cell (ESC)-specific gene expression by promoting crosstalks among multiple distal and proximal enhancers with promoters^8^. Hence, while ChIP-sequencing is classically used to define CBP or P300 binding repertoires, it is a poor proxy to identify the gene networks controlled by these two factors and to characterise their biological activities.

While CBP and/or P300 were shown in cell-based assays to act as co-activators for several transcription factors critically involved in myogenesis, such as MyoD, Myogenin or Mef2C^9–12^, their respective function in this process remains poorly characterised. Vertebrate myogenesis requires the cooperation of the myogenic regulatory factors (MRFs) MyoD, Myf5, Mrf4 and Myogenin^13^, and the members of the Myocyte-enhancer factors 2 (Mef2) family, with chromatin remodelling or modifying enzymes (for reviews,^14–17^. Myoblast differentiation is a sequential process that starts by the withdrawal from the cell cycle, followed by the expression of factors involved in the muscle specific transcriptional program. Thereafter, myoblasts undergo morphological changes by first elongating, before fusing into multinuclear myofibers. Hence, during this process, myoblasts undergo dramatic changes in morphology and gene expression.

To date, most studies aimed at analyzing the impact of CBP or P300 on myogenesis used C2C12 cells, an immortalised cell line derived from mouse muscles, and focused on the expression of a handful of myogenic factors^9–12^. However, the respective role(s) of CBP and P300 during myogenesis has neither been addressed at global scale or in humans. Therefore here we used human primary myoblasts to characterise the phenotypes associated with CBP and/or P300 knockdown and to identify the gene networks regulated by these two co-activators.

We found that the majority of the deregulated genes are divergent between CBP and P300, showing for the first time the respective consequences of their downregulation at genome-wide expression level in the differentiation of human primary myoblasts. Our data also indicate that CBP and P300 are complementary to each other by acting on distinct genes belonging to the same pathways. In particular, we found that besides their requirement for the activation of the muscle gene expression program, they regulate the expression of various extracellular signalling pathways, cell cycle factors or replication complexes. Interestingly, they also concur to the expression of different cell adhesion complexes, including components of the dystrophin-glycoprotein complex and the α7β1 integrin complex, which are essential for the maintenance of skeletal muscle integrity and are mutated in human muscle disorders (for a review^18^). Accordingly, P300 as well as CBP were both required for muscle cell attachment. This finding was further validated using zebrafish as a model system in which decreased CBP and P300 HAT activity led not only to the downregulation of the homologous genes implicated in ECM/muscle interaction but also to a dystrophic-like phenotype. Our results thus indicate that CBP and P300 are both crucial for proper adult myogenesis in human and act primarily *via* distinct though converging gene networks. Finally, we propose that modulation of CBP and P300 activity could be implicated in muscular dystrophy.

## Results

### CBP and P300 knockdowns affect human primary myoblast differentiation

**Figure 1:**
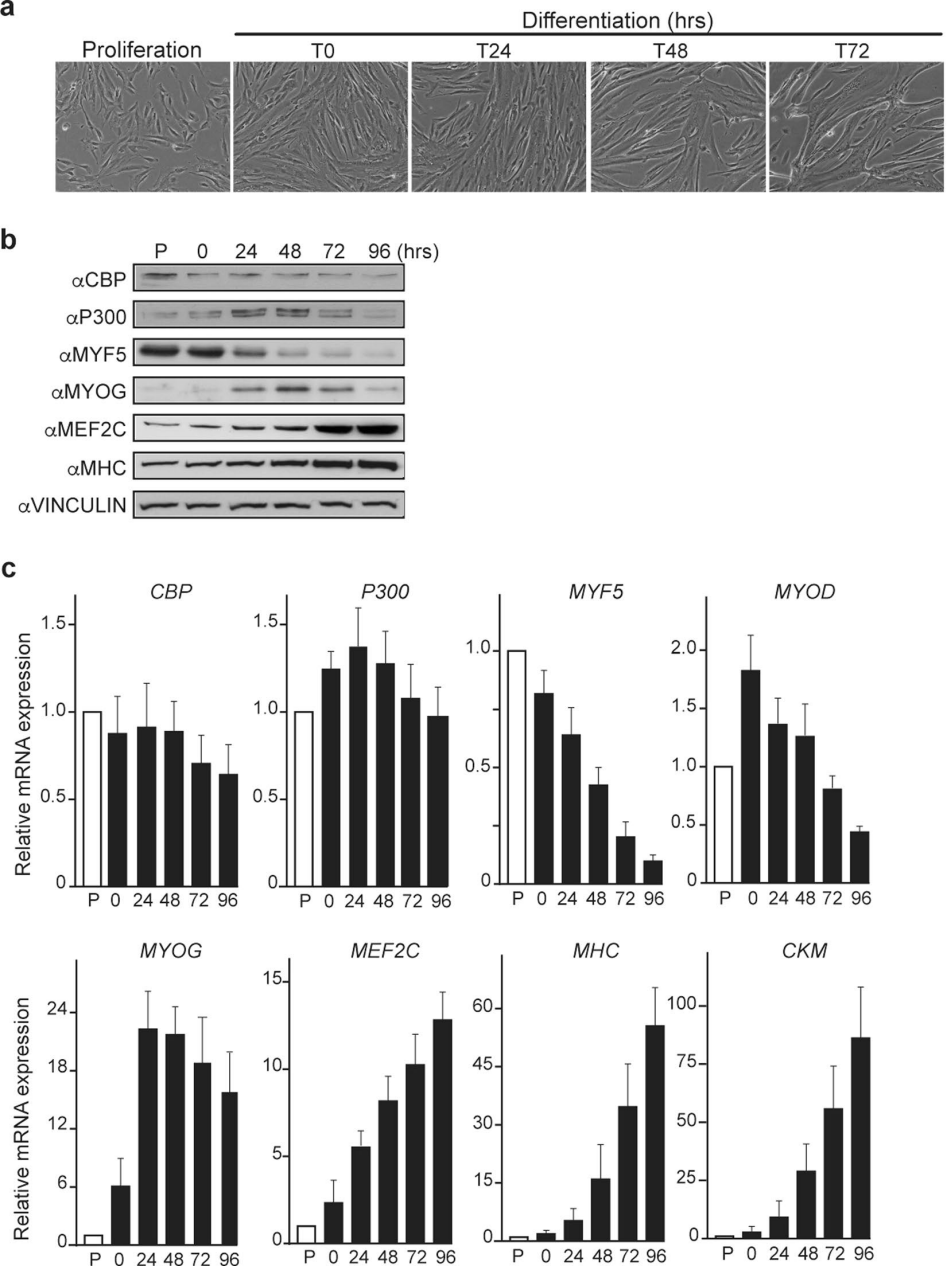
Human Primary myoblasts (HPMs) differentiate into myotubes and properly express myogenic factors. (**a**) Phase contrast photographs of proliferating HPMs, HPMs at the induction of differentiation (T0), HPMs after 24 (T24), 48 (T48) and 72 (T72) hours of differentiation. (**b**) Western blots of HPM cell extracts in proliferation (P) or at the indicated differentiation time points with antibodies recognizing CBP, P300, the myogenic factors/markers MYF5, MYOGENIN, MEF2C and MHC as well as the loading control VINCULIN. (**c**) Relative expression of *CBP*, *P300*, *MYOD*, *MYOGENIN (MYOG)*, *MEF2C*, *MHC*, *CKM* transcripts to *GAPDH* expression as measured by quantitative PCR in proliferating HPMs or in HPMs at different time points of differentiation as indicated. Graphs show mean ± SD values from three independent experiments.

Human primary myoblasts (HPMs) were used to study the respective involvement of CBP and P300 in myogenesis. HPM differentiation can be induced by serum starvation when cells reach confluence and this process is accompanied by striking phenotype changes: at 24 h after induction of differentiation, cells elongate, then align to each other at around 48 h to finally fuse to form myotubes from 72 h onwards (Fig. 1a). To monitor this process, we also assessed the expression of several molecular markers by Western blot and by RT-qPCR (Fig. 1b,c). Specifically, expression levels of the early myogenic transcription factors MYF5 and MYOD decreased during differentiation while expression of the later myogenic factors MYOG and MEF2C or of the differentiation markers Myosin Heavy Chain (MHC) and Creatine Muscle Kinase (CKM) was strongly enhanced during this process (Fig. 1b,c). CBP and P300 were both expressed in proliferating and differentiating myoblasts but exhibited different dynamics *i.e*. while CBP expression decreased during differentiation both at protein and RNA levels, P300 protein and transcript showed a transient increase at 24 h and 48 h (Fig. 1b,c).

**Figure 2:**
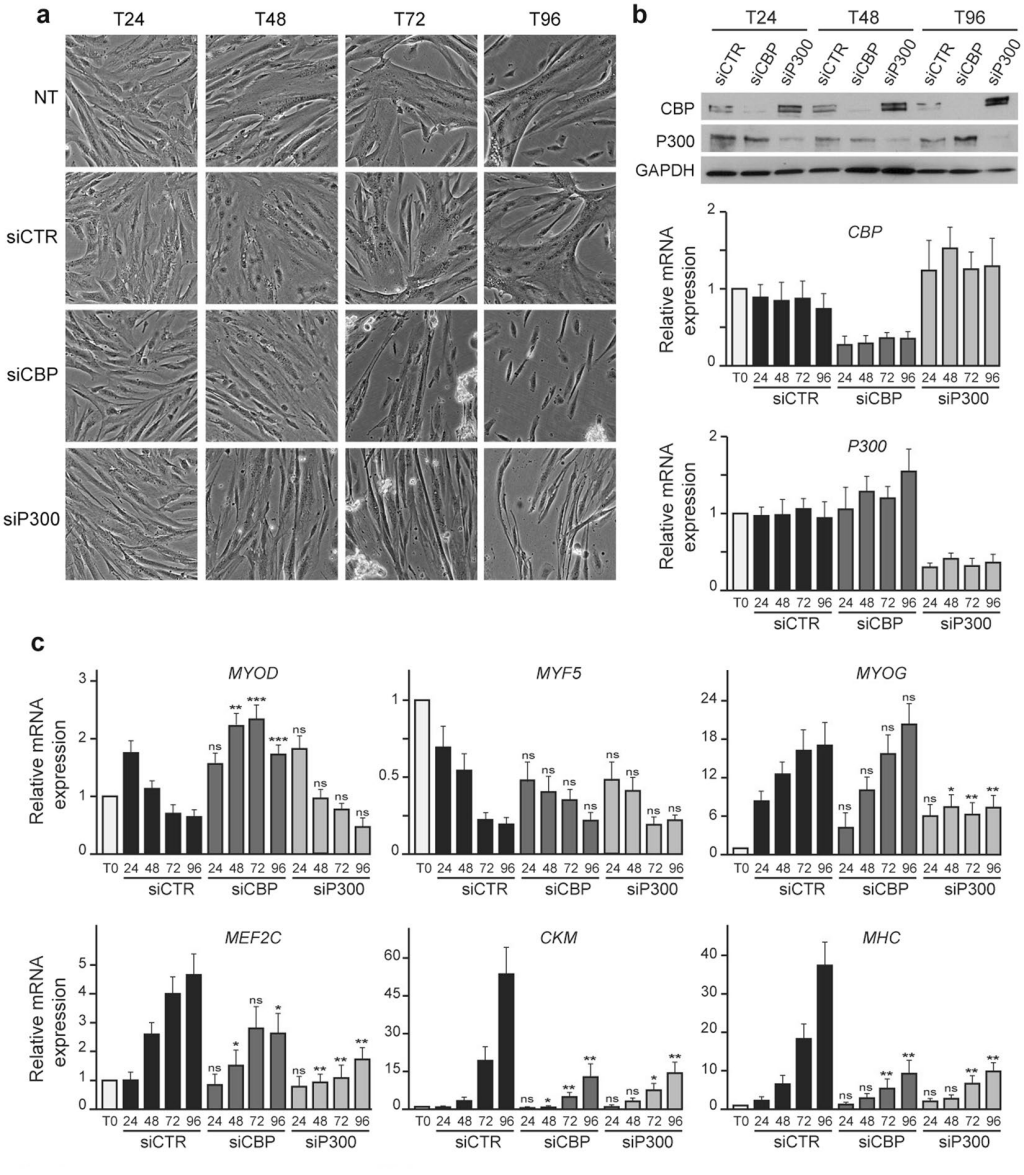
Impact of CBP or P300 knockdown on HPM differentiation and on myogenic factor expression. (**a**) Phase contrast photographs of non-transfected HPMs (NT) or HPMs transfected with either a control siRNA (siCTR) or siRNAs directed against CBP (siCBP) or against P300 (siP300) after different times of transfection 24 h (T24), 48 h (T48), 72 h (T72) and 96 h (T96), corresponding to 0 h, 24 h, 48 h and 72 h of differentiation, respectively. (**b**) Upper panel: western blot showing the expression of CBP and P300 at different times of transfection as indicated after knockdown of CBP or P300. GAPDH was used as loading control. Lower panels: quantitative PCR analyses showing the expression of *CBP* and *P300* transcripts relative to *GAPDH* expression in proliferating HPMs (T0) or in HPMs after siCTR, siCBP or siP300 transfection, at different time points after transfection as indicated. Graphs show mean ± SD values from at least three independent experiments (lower panel). (**c**) Relative expression of *MYOD, MYF5, MYOG, MEF2C, CKM* and *MHC* transcripts to *GAPDH* expression by quantitative PCR in proliferating HPMs (T0) or in HPMs after siCTR, siCBP or siP300 transfection, at different time points of transfection as indicated. Graphs show mean ± SD values from at least three independent experiments. Statistical significance are relative to siCTR and were calculated with an unpaired *t-test*; ns: not significant; *p < 0.05, **p < 0.01.

In order to assess the function of CBP and P300 in myogenic differentiation, sub-confluent HPMs were transfected with either control or specific siRNAs directed against *CBP* or *P300* mRNAs (hereafter called siCBP and siP300) before induction of differentiation by serum starvation. The phenotypes of HPMs transfected with the various siRNAs were analyzed at different time points. While cells transfected with control siRNAs underwent myogenic differentiation similarly to non-transfected (NT) cells, those transfected with either siCBP or siP300 showed defects especially from 72 h onwards post-transfection *i.e*. 48 h after differentiation induction (Fig. 2a). Indeed, following *CBP* or *P300* knockdown, HPMs elongate, align with each other and form myotubes/myofibers but detach thereafter from the petri dish when the first contractions are observed so that at late time of differentiation only a minority of differentiated cells remain attached (Fig. 2a). *CBP* and *P300* knockdowns were confirmed by Western blot and RT-qPCR, each siRNAs specifically affecting the expression of its target with a slight compensatory effect between *CBP* and *P300* (Fig. 2b). Of note, similar phenotypes and effects were observed using two other sets of siRNAs directed against *CBP* or *P300* mRNA (Supplementary Fig. S1a,b). In parallel, we assessed by RT-qPCR whether the expression of key myogenic transcription factors and differentiation markers was affected (Fig. 2c). Both siCBP and siP300 impaired the increase of *MEF2C*, *CKM* and *MHC* expression at late times of differentiation. In contrast, they had distinct impacts on the expression of *MYOD*, which was increased by siCBP but not affected by siP300, and of *MYOG*, which was downregulated by siP300 only (Figs 2c and S1c). Importantly, similar results were obtained after CBP or P300 knockdown in HPMs purified from biopsies from two independent donors, indicating that the observed effects were not specific of a given donor but reflect the genuine function of CBP an P300 in HPM differentiation (Supplementary Fig. S2). In sum, these results indicate that HPMs deprived of CBP or P300 initiate myogenesis but do not progress normally toward differentiation and ultimately show strong adhesion defects. These data also suggest that CBP and P300 regulate differentially myogenesis in human.

### CBP and P300 are critical for the regulation of distinct gene subsets

**Figure 3:**
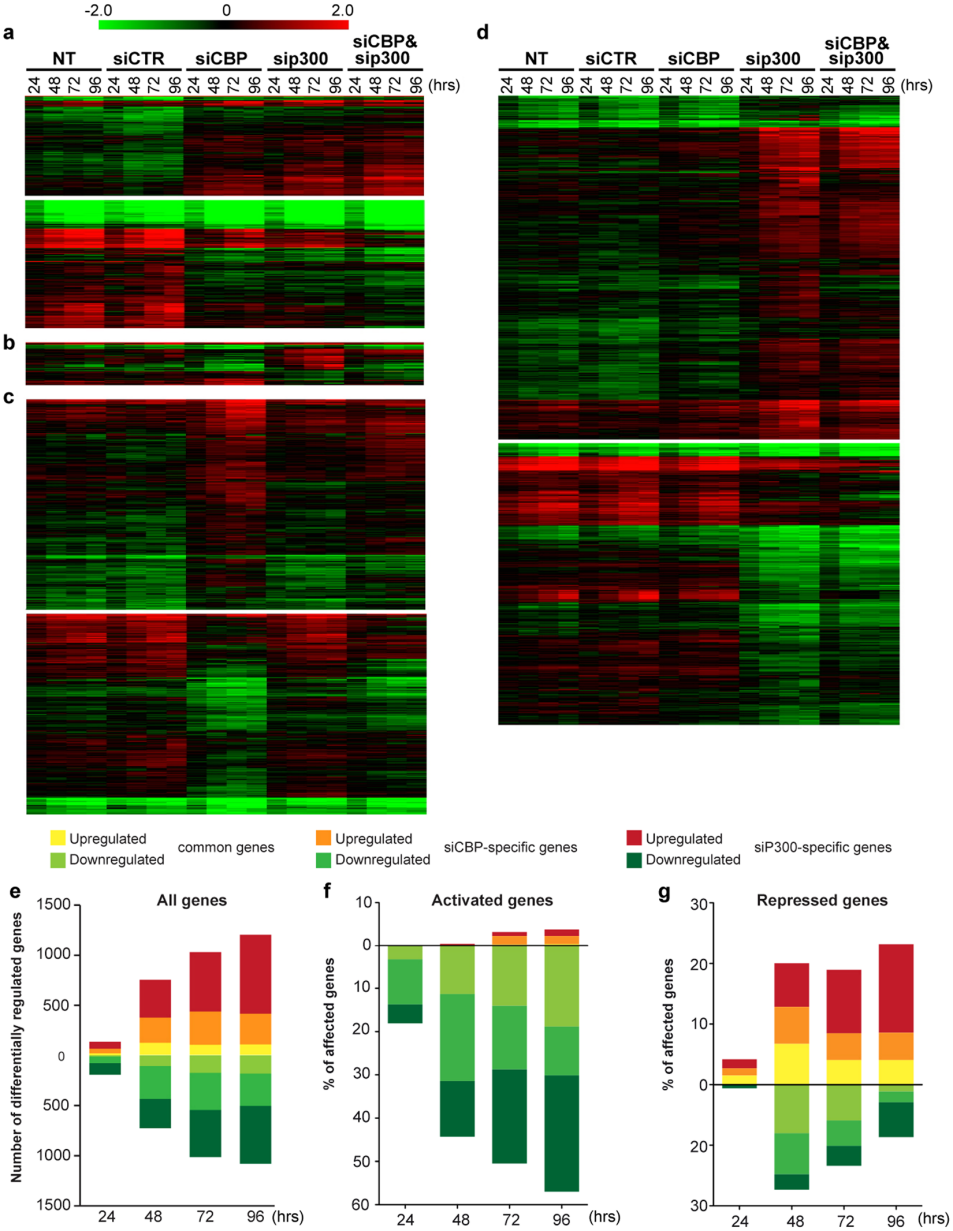
CBP and P300 regulate common and divergent gene subsets. Heatmaps of (**a**) genes down- or upregulated by CBP and P300, (**b**) genes regulated in an opposite way by CBP and P300, (**c**) genes specifically down- or upregulated by CBP, (**d**) genes specifically down- or upregulated by P300. (**e**) Number of genes down- or upregulated by either CBP or P300 or both at different transfection time points. (**f**,**g**) Proportion of genes activated (**f**) or repressed (**g**) during HPM differentiation that are affected by CBP or P300 knockdown.

To gain insights into the common and divergent involvement of CBP and p300 in the gene regulation underlying myogenesis, we compared at four time points of HPM differentiation the transcriptomes of cells in which CBP, P300 or both were knocked-down by siRNA, with that of control cells. The lists of genes specifically regulated by CBP and/or P300 were generated using a threshold of 1.8 fold change relative to the corresponding time point of siRNA control (siCTR)-transfected HPMs (Supplementary Tables S1). Transcriptomics data were validated by RT-qPCR for a number of transcripts (Supplementary Fig. S3). We found that the expression of more than 3,000 genes was altered for at least one of the four time points after induction of differentiation in cells transfected with either siCBP or siP300 (Supplementary Tables S1). Differentially expressed genes were then classified to discriminate the effect of CBP and P300 on gene expression according to the following criteria: upregulation *versus* downregulation and common *versus* specific for CBP or P300 (Supplementary Table S2). These lists were further used to build heatmaps at all time points of differentiation, illustrating the different categories defined by the above criteria (Fig. 3a–d). As outlined from these figures, CBP and P300 mostly regulate distinct gene sets: 1,617 (48%) genes were only affected by P300 (Fig. 3d) and 1,048 (31%) were specific to CBP (Fig. 3c), while 623 (19%) genes were commonly affected by both factors (Fig. 3a) and 76 (2%) were regulated in an opposite manner (Fig. 3b). Analyzing the number of genes affected at each time point confirmed that a high proportion of genes was affected specifically by each HAT (Fig. 3e). Indeed, around 75% of the genes regulated by P300 were not shared by CBP and *vice versa*, and this was true independently of the time point considered and if the genes were up- or downregulated during differentiation (Fig. 3e). These results support the idea that CBP and P300 are redundant for the regulation of a small set of genes, but that the major fraction of genes requires the selective action of one of these HATs.

To more specifically assess the effect of CBP and P300 on the myogenic differentiation program, we also monitored the effect of CBP and P300 knockdown on the genes that are either activated or repressed during normal differentiation (Fig. 3f,g and Supplementary Tables S1). This revealed that, at each time point of differentiation, 18 to 60% of the activated genes and 5 to 32% of the repressed genes were not properly expressed following CBP or P300 knockdown. Thus, in aggregate, both factors are required for the proper expression of ± 40% of genes whose expression is modified during HPM differentiation. Of note, CBP and P300 knockdown primarily impaired the upregulation of the genes activated during myogenesis, while it caused both activation and downregulation of the repressed genes. Hence, CBP and P300 play a critical role in the activation of the human myogenic program.

Next, we compared the genes deregulated by CBP or P300 single knockdown to those affected in the double knockdown. Between 52 and 72% and between 38.5% and 62.5% of the genes downregulated in either of the single knockdown were also repressed by the double knockdown at any time point (Supplementary Fig. S4a). Similarly, 52 to 73% and 44 to 56% of the genes activated in either siP300 or siCBP were commonly upregulated in the double knockdown (Supplementary Fig. S4b). Thus, the double knockdown recapitulates the majority of the deregulations observed in the single knockdown. Conversely, 35 to 45% or 50 to 59% of the genes respectively repressed or activated in the double knockdown were not affected in either of the two single knockdowns, suggesting that their expression is regulated by CBP and P300 in a redundant manner (Supplementary Fig. S4a,b). Moreover, consistent with the results obtained with the single knockdowns (Fig. 3e,f), the double knockdown compromised the expression of respectively 22 to 68% and 6 to 26% of the genes activated or repressed at the different time points of HPM differentiation (Supplementary Fig. S4c). Thus, these data confirm the key role of CBP and P300 in the rewiring of gene expression during muscle differentiation and identify a gene population for which CBP and P300 may be functionally redundant.

### CBP and p300 regulate different components of major cellular processes

To reveal the key biological processes controlled by CBP and/or P300 in HPMs, gene ontology (GO) analyses were performed with all the genes downregulated or upregulated after CBP or P300 knockdown (Supplementary Table S3). A selection of the most representative top-scoring GO categories is presented Table 1. In addition, to better define the specific and common functions of these two HATs, we built gene networks from the lists of genes that are specifically downregulated or upregulated by siCBP or siP300 and that are commonly deregulated by siCBP and siP300 (Supplementary Fig. S5).

**Table 1:** Gene ontology (GO) analysis of the genes down- or upregulated by the CBP or P300 single knockdown or by the double knockdown CBP&P300.

GO-Term	GO-Term ID	All siCBP down	All siP300 down	Specific siCBP down	Specific siP300 down	Common siCBP & siP300 down	All double siRNA down	Specific double siRNA down
**Muscle structure development**	**GO:0061061**	**1.42E-12 (69)**	**5.25E-23. (96)**	**8.01E-04 (30)**	**2.63–12 (51)**	**1.02E-11 (39)**	**6.28E-38 (142)**	**8.32E-09 (37)**
**Actin filament-based process**	**GO:0030029**	**1.35E-07 (61)**	**9.22E-11 (76)**	**2.76E-04 (34)**	**2.91E-07 (49)**	**1.14E-04 (27)**	**7.98E-24 (125)**	**8.11E-07 (35)**
**Mitotic cell cycle**	**GO:0000278**	**1.19E-09 (86)**	**1.34E-09 (95)**	**n.s. (36)**	**n.s. (45)**	**3.01E-11 (50)**	**6.88E-09 (118)**	**n.s. (23)**
**Chromosome segregation**	**GO:0007059**	**7.36E-14 (50)**	**4.67E-11 (49)**	**n.s. (13)**	**n.s. (12)**	**1.33E-18 (37)**	**1.36E-06 (49)**	**n.s. (4)**
**Sarcomere organization**	**GO:0045214**	**1.07E-04 (9)**	**5.90E-15 (20)**	**n.s. (2)**	**9.66E-10 (13)**	**1.22E-05 (7)**	**1.12E-20 (27)**	**2.73E-04 (6)**
**Cholesterol biosynthetic process**	**GO:0006695**	**4.71E-11 (19)**	**n.s. (4)**	**1.24E-11 (16)**	**n.s. (1)**	**n.s. (3)**	**2.80E-09 (21)**	**n.s. (2)**
**Extracellular matrix organization**	**GO:0030198**	**7.91E-04 (28)**	**1.76E-04 (33)**	**n.s. (15)**	**n.s. (20)**	**n.s. (13)**	**1.80E-15 (67)**	**6.32E-09 (26)**
***GO-Term***	***GO-Term ID***	***All siCBP up***	***All siP300 up***	***Specific siCBP up***	***Specific siP300 up***	***Common siCBP & siP300 up***	***All double siRNA up***	***Specific double siRNA up***
*Type I interferon signalling pathway*	*GO:0060337*	*n.s. (5)*	*3.23E-10 (23)*	*n.s. (3)*	*1.46E-10 (21)*	*n.s. (2)*	*6.76E-10 (26)*	*n.s. (5)*
*Positive regulation of gluconeogenesis*	*GO:0045722*	*2.07E-05 (6)*	*n.s. (1)*	*2.53E-06 (5)*	*n.s. (0)*	*n.s. (1)*	*n.s. (1)*	*n.s. (1)*
*Regulation of RNA biosynthetic process*	*GO:2001141*	*1.57E-05 (172)*	*n.s (234)*	*9.29E-04 (110)*	*n.s. (172)*	*n.s. (62)*	*5.12E-10 (372)*	*3.29E-04 (148)*

Top-scoring most representative GOs related to downregulated genes by CBP, P300 or CBP&P300 are in bold, while GOs associated with upregulated genes are in italic and underlined. P-values are indicated (“n.s.”: not significant/p-value ≥ 10^−3^) Numbers into brackets represent the absolute number of genes.

Concerning the upregulated genes, we observed limited GO enrichments and basically no overlap between the processes deregulated by CBP or P300 knockdown. Still, siP300 was associated with the induction of genes implicated in nucleosome assembly or type I interferon signalling pathway, and siCBP with the induction of genes participating in gluconeogenesis (Table 1). Correspondingly, two of the main hubs present in siCBP upregulated gene network, *PPARA* and *PPARGC1A*, are involved in gluconeogenesis^19,20^ (Supplementary Fig. S5a). Moreover, several hubs in the different upregulated gene networks correspond to genes associated with inflammatory/immune response; for instance *STAT1*^21^, *EDN1*^22^ or *IL15*^23^ in siP300, *CCL2*^24^ or *NOTCH1*^25^ in siCBP, and *CXCL12*^24^, *EGFR*^26^, *FGF2*^27^ or *TLR3*^28^ in siCBP and siP300 (Supplementary Fig. S5a). These data suggest that a reduction of P300 and/or CBP could trigger inflammatory conditions. Of interest, the impact of CBP or P300 seemed independent of apoptosis as no apoptotic gene was upregulated and no GO enrichment related to apoptotic pathways could be detected in CBP or P300 knockdown conditions (Supplementary Table S3).

Concerning the downregulated genes, we observed a strong over-representation of GO categories related to muscle development upon CBP or P300 knockdown, which confirmed the critical role of these two HATs in the activation of the human myogenic program (Table 1). In addition, both HATs are required for the proper expression of genes implicated in mitotic cell cycle or chromosome segregation, as well as in actin filament-based process or extracellular matrix adhesion. Of note, most of the top scoring GO terms were associated with both siCBP and siP300, whereas only a few GO categories were specifically associated with CBP or P300 knockdown (*e.g*. sarcomere organisation with siP300, cholesterol metabolism with siCBP). Thus, CBP and P300 knockdowns essentially lead to the upregulation of genes participating in the same biological processes.

**Figure 4:**
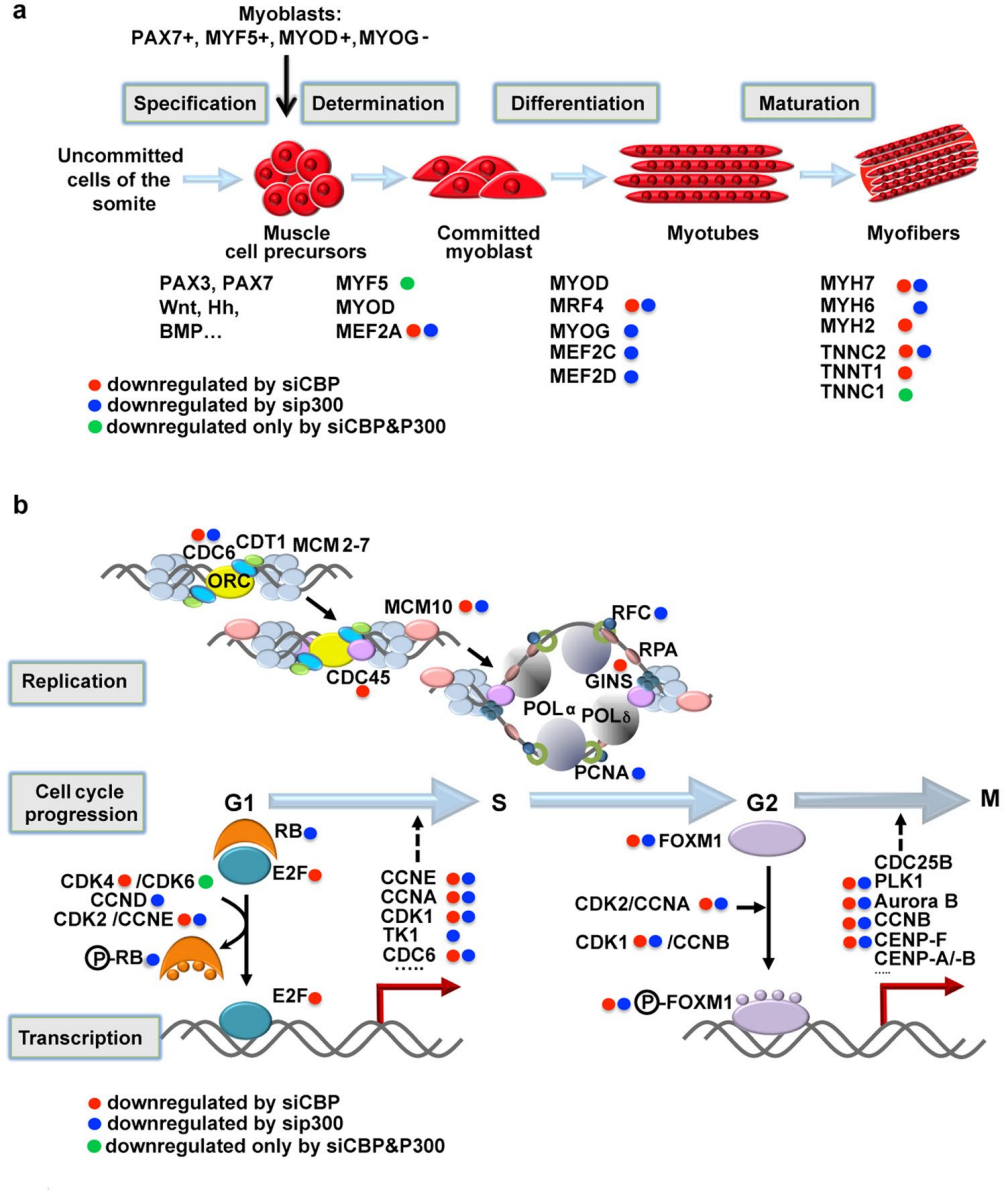
Impact of CBP and P300 knockdown on myogenesis, replication and cell cycle regulation. (**a**) Simplified schema showing the different steps of myogenesis and the impact of CBP and/or P300 on the corresponding myogenic factors/markers. Schema adapted from^15^. (**b**) Schematic representations of DNA replication and the factors under the control of CBP and/or P300, after^77^ (upper panel) and of cell cycle progression, after^78^ (lower panel). (**a**,**b**) Red dots, blue dots and green dots label factors whose transcripts are downregulated by CBP, P300 or the double CBP&P300 knockdown, respectively.

Interestingly, besides the genes commonly regulated by these two HATs in these processes, an important fraction of the genes was specifically affected by one or the other (Table 1). For example, out of 61 genes downregulated by siCBP that participate in actin filament organisation, 35 are specific to CBP and 26 are common with P300. More specifically, among the myogenic factors/markers, *MEF2A*, *MYF6/MRF4*, *MYOSIN HEAVY CHAIN* 7 *(MYH7*) or *TROPONIN C*2 (*TNNC2*) were regulated by both CBP and P300, *MYOG*, *MEF2C*, *MEF2D or MYH6* were specifically under the control of P300, and *MYH2* or *TNNT1* were specifically regulated by CBP (Fig. 4a). Of note, the Early Growth Response 1 (EGR1) transcription factor appearing as an important hub in the gene network downregulated by siP300 (Supplementary Fig. S5b). EGR1 having been shown recently to induce myogenic differentiation by activating *Myogenin* expression^29^, our data identify this factor as a new key player in muscle differentiation. Similarly to myogenic factors, CBP and P300 seem to act in a complementary manner to control components of the replication machinery: CBP controls *CDC45* and the *GINS* (Go-Ichi-Ni-San) complex, while P300 regulates *RFC* (Replication Factor C) and *PCNA* (Proliferating Cell Nuclear Antigen) (Fig. 4b). For cell cycle progression, CBP controls *E2F1* and *CDK4* expression while P300 regulates *RB* and *CYCLIN D* leading in both cases to the regulation of E2F target genes and thus to the G1/S transition arrest. Moreover, both CBP and P300 inhibit the G2/M transition by regulating *FOXM1*, *CYCLIN A* and *CDK1* expression (Fig. 4b). Consistent with a key role for these two HATs in the control of cell cycle progression, *E2F1/CDK4, RB1* and *CDK1/FOXM1* appeared as important hubs in the downregulated gene networks of siCBP, siP300 and of both siCBP and siP300, respectively (Supplementary Fig. S5).

**Figure 5:**
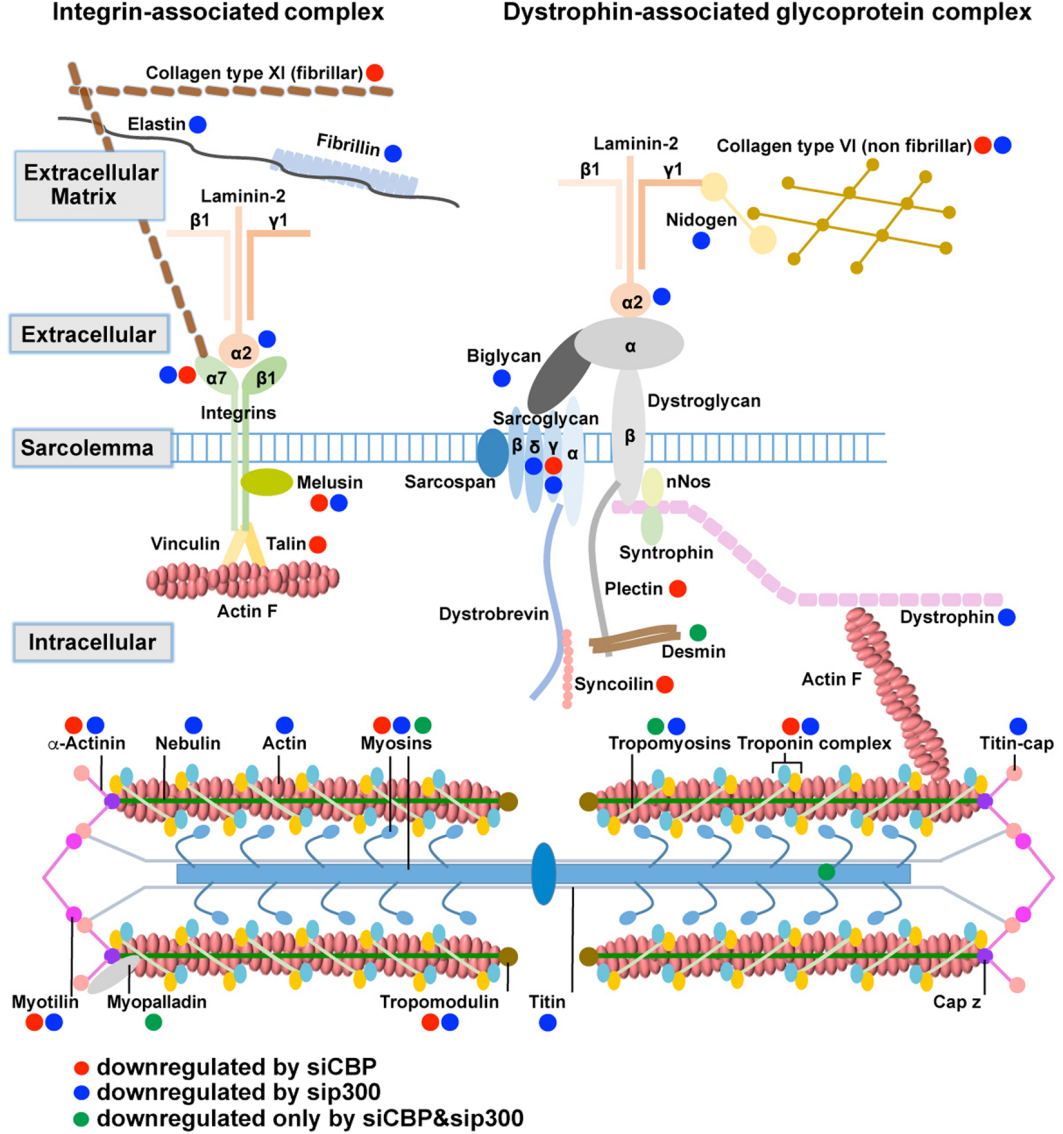
Impact of CBP and P300 knockdown on cell adhesion. CBP and P300 regulate the expression of proteins belonging to the integrin- and dystrophin-associated glycoprotein complexes, from the extracellular matrix to the sarcomere. Red dots, blue dots and green dots label factors whose transcripts are downregulated by CBP, P300 or the double CBP&P300 knockdown, respectively. Adapted from^79–81^.

Our analyses also revealed that CBP and P300 are required for the proper expression of several factors implicated in cell adhesion, notably within the Dystrophin-Glycoprotein complex and the α7β1 Integrin complex, which are often deregulated in human dystrophies (Fig. 5) (for a review^30^). While both CBP and P300 regulate the expression of sarcomeric proteins like MYH7, Tropomodulin, Troponin C2 (TNNC2), Myotilin, α-Actinins and of a number of proteins belonging to the upstream complexes such as Integrin α7, Melusin, or Sarcoglycan γ, they also regulate distinct genes. This is the case for *Collagen type XI, Talin, Plectin, Syncoilin, MYH6, Troponin T1* for CBP while P300 regulates among others, *Nebulin, Elastin, Fibrillin, Laminin α2, Biglycan, MYH6, Sarcoglycan δ* and *Dystrophin* (Fig. 5). Hence, by impairing the expression of these factors, CBP and P300 knockdowns could lead to the development of dystrophic phenotypes.

In sum, it appears that CBP and P300 act on the same processes in part by controlling complementary sets of genes. Our results also point out that the role of CBP and P300 in human primary myoblast/myotube extends beyond the control of their differentiation and encompasses the control of key biological functions.

To analyse whether these different processes might be directly regulated by CBP and/or P300, we crossed our datasets with that of Blum *et al*. in which they identified in mouse C2C12 cells genome-wide active enhancers and their associated genes on the basis of ChIP-seq data with p300, H3K4me1, H3K18ac, H3K27ac –an acetylation mark specifically deposited by CBP and P300- and polII^31^. Thus, these enhancers and associated genes are likely direct targets of CBP and/or P300 during myogenic differentiation^31^. Thereby we could define a list of enhancers/genes that are likely direct targets of P300 and/or CBP (since they are bound by P300 and/or contain a histone mark deposited by CBP or P300) in mouse C2C12 cells undergoing myogenic differentiation. Crossing this list of enhancer associated genes with our datasets indicated that among the downregulated genes following siCBP or siP300 treatment in HPM, a proportion of 20.6% and 24.6% might represent genes directly activated by CBP or P300, respectively (Supplementary Table S4). Interestingly, GO analyses of these “direct” genes revealed that muscle structure development, actin filament-based process, cholesterol biogenesis and adhesion processes are significantly enriched and thus, most likely directly regulated by CBP and/or P300, while cell cycle regulation, chromosome segregation and sarcomere organization are probably regulated indirectly by the two coactivators. On other hand, the same analyses performed with genes upregulated upon siCBP or siP300 did not show any significant enrichment in GO related to inflammation, gluconeogenesis or RNA biogenesis (Supplementary Table S4). Thus, these upregulated processes are most likely controlled indirectly by CBP and P300. Indeed, a lot of late-time changes in gene expression and especially upregulated genes might be due to the cascade effect of genes differentially regulated at earlier times as bona fide targets of CBP/p300 inhibition.

### CBP and P300 are redundant for gene subsets

Next, we focused on the genes that were only deregulated following P300 and CBP double knockdown and that likely reflect the redundant role of these HATs. Besides GO categories already enriched in either of the single knockdown such as muscle structure development (Table 1), siCBP&siP300-dependent downregulated genes were most strongly associated with extracellular matrix organisation and cell adhesion (Table 1), suggesting that the level of redundancy between CBP and P300 in HPMs is particularly strong for these two processes (Fig. 5). Notably, CBP and P300 are redundant for the expression of at least 6 collagens, 10 matrix metalloproteinases (MMPs), 3 intercellular adhesion molecules (ICAMs) and 2 integrins (Supplementary Table S3). Moreover, the gene regulatory network downregulated after the double knockdown siCBP&siP300 highlighted *MYC* and *AGT* as two prominent hubs (Supplementary Fig. S6a). MYC has been identified as a major factor that is responsible for the synthetic lethality observed in *CBP*-deficient cells after ablation of *P300*^32^. Similarly, *AGT* expression has been shown to require both CBP and P300^33^. These observations reinforce the validity and the interest of our knockdown approach.

Concerning the genes solely upregulated after the double knockdown of CBP and P300, no strong GO enrichment was observed (Tables 1 and S3). Nevertheless, the corresponding gene network (Supplementary Fig. S6b) identified several hubs of genes that are involved in apoptosis, like *BAX* (*Bcl2-Associated X*)^34^, *miR-34A*^35^ and *PRKCD* (*Protein Kinase C Delta*)^36^ or in innate immunity/inflammation such as *IKBKE (Inhibitor of Nuclear Factor Kappa B Kinase Subunit Epsilon*)^37^ and *CEBPD* (*CCAAT/Enhancer Binding Protein Delta*)^38^. HDAC1 also came out as a major hub and could in this context, either trigger cell cycle arrest and senescence^39^ or be involved in inflammation^40^.

These data underscore the redundant role of CBP and P300 in promoting cell survival as well as in the regulation of muscle cell attachment.

### Decreased CBP and P300 HAT activities in Zebrafish leads to a muscular dystrophy-like phenotype

To analyze *in vivo* whether and to which extend CBP and P300 regulate muscle adhesion/attachment/integrity, we made use of the zebrafish model, which has become instrumental to address whether the loss of components contributes to muscle physiology and pathology^41^. Indeed, zebrafish dystrophic mutants and morphants with skeletal muscle abnormalities represent informative models of muscle disease pathogenesis and therapy^41–43^. In most of these mutants, skeletal muscle development appears normal but the first contractions trigger muscle cell detachment from their insertion site, which is followed by myotome degeneration^42^. Accordingly, the detachment of muscle fibers is a common feature of several dystrophic mutants and/or morphants.

**Figure 6:**
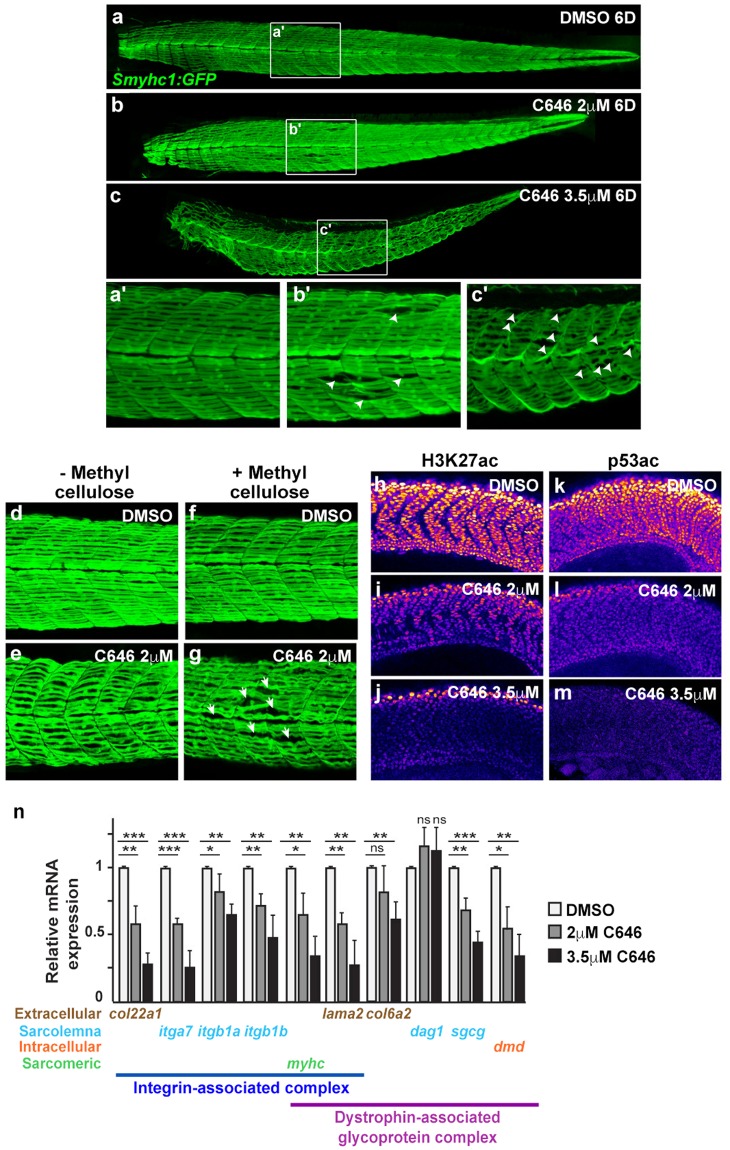
P300/CBP histone acetyltransferase activity inhibition induces muscle cells detachment in zebrafish. (**a–c**) Confocal projections of transgenic Tg*(smyhc1:EGFP)*^*i104*^ embryos treated with either DMSO (vehicle), 2 μM or 3.5 μM C646 at 10 hpf for 24 h, fixed and imaged at 6 dpf. (a’–c’): higher magnification views of the boxed area in **a–c**. Arrowheads indicate muscle cell attachment abnormalities. (**d–g)** Confocal projections of transgenic Tg*(smyhc1:EGFP)*^*i104*^ embryos treated with either DMSO or 2 μM C646 from 10 hpf for 24 h, and further raised in water (−Methylcellulose; **d**,**e**) or in viscous medium (+Methylcellulose; **f**,**g**) until 5 dpf. (**h–m**) Immunostaining against either H3K27ac (**h–j**) or p53ac (**k–m**). Confocal sections of embryos treated with either DMSO (vehicle), or 2 μM or 3.5 μM C646 at 10 hpf for 10 h and fixed at 20 hpf, as indicated on the panels. Fluorescent intensity is color-coded. (**n**) Relative mRNA expressions were determined by RT-qPCR in 3 dpf embryos treated with either DMSO, or 2 μM or 3.5 μM C646 from 10 hpf for 24 h. Error bars represent S.D. Statistical significance is relative to control and was calculated with an unpaired *t-test*. ns: not significant; *p < 0.05, **p < 0.01, ***p < 0.001. A minimum of 12 embryos were analysed per condition.

To inhibit CBP and P300 HAT activities in fish, embryos were treated with increasing concentrations of C646, a specific CBP/P300 HAT inhibitor^44^, and the resulting phenotypes were analyzed. We found that the addition of C646 resulted in a muscular dystrophy-like phenotype at 6 days of development, *i.e*. embryos showed a trunk/tail curvature concomitant with muscle fiber detachment (Fig. 6a–c). Moreover, the addition of methylcellulose to increase water viscosity led to a more dramatic detachment/disorganization of the somites in zebrafishes exposed to a low amount of C646, further validating the dystrophic nature of the phenotype (Fig. 6c–g). This phenomenon was not due to apoptosis as we did not detect any increase of cleaved caspase 3 expression by immunofluorescence (Fig. S8), and the expression of the anti-apoptotic gene *bcl2* or of the pro-apoptotic genes *bax* and *puma* was not affected upon CBP/P300 inhibition, as revealed by RT-qPCR (Fig. S8). Of note, CBP/P300 HAT activity was efficiently reduced in these conditions as assessed by the dose-dependent decrease of H3K27 and p53 acetylation, two well-established substrates of CBP and P300^45^ (Fig. 6h–m).

Finally, we checked by RT-qPCR whether the inhibition of P300 and CBP in zebrafish also affected the expression of the integrin- and dystrophin-associated adhesion complexes as observed in human primary myoblasts (Fig. 6n). Strikingly, all the genes tested that were downregulated in HPMs following CBP and/or P300 knockdown, were also repressed in C646-treated zebrafish. Moreover *dystroglycan* (*dag1*), whose expression was not affected by either CBP or P300 in human cells (Fig. 5), was not deregulated either by C646 in zebrafish (Fig. 6n). Altogether these data indicate a strong impact of CBP and P300 on myogenic cell adhesion in human cells and in zebrafish and suggest that a reduction in the enzymatic activity of these co-activators leads to a dystrophic phenotype in zebrafish and potentially in human cells.

## Discussion

Here, we assessed and compared the role of CBP and P300 during myogenesis at a global scale in human primary myoblasts. In sum, our data show that these two related factors control human myogenesis by regulating essentially distinct gene networks and highlight an unforeseen link between CBP/P300 activity and the emergence of dystrophic phenotypes.

To characterise CBP and P300 function in myogenesis, we used an siRNA-mediated knockdown strategy in human primary myoblasts. SiRNA approach was chosen rather than CRISPR/CAS9 as the knockout of *CBP* or *P300* inhibited cell proliferation and triggered cell death by mitotic catastrophe^46^. In addition, knowing the limitations of ChIP-sequencing data with CBP and P300 and in particular the poor correlation between CBP/P300 binding and expression of the nearest gene^7^, we did not look for direct CBP or P300 target genes but rather assessed at global scale the transcriptional or post-transcriptional consequences of CBP and/or P300 knockdown using microarrays. However, merging our data with genes associated with active enhancers -that bound P300 or were enriched in H3K27 acetylation mark and could thus be direct targets of CBP and/or P300- identified previously in C2C12 cells^31^ indicated that while processes such as myogenic differentiation, actin filament formation, cholesterol metabolism and adhesion complex were most likely regulated directly by CBP and/or P300, upregulated pathways were probably a consequence of the cascade effect of genes differentially regulated at earlier times as bona fide targets of CBP and/or p300 inhibition.

Due to their high sequence homology^47^, these two proteins have often been referred to as CBP/P300 since their identification, and their activity has commonly been considered as largely redundant. By comparing the knockdown of each of these two factors and their concomitant knockdown, we bring new insights into the redundant and the divergent function(s) of CBP and P300. Our results clearly show that these two factors chiefly regulate distinct gene sets to control several aspects of HPM biology, even though they also exhibit some degree of redundancy.

Our phenotypic analysis indicates that the knockdown of CBP or P300 is not sufficient to prevent the early steps of myogenic differentiation (*i.e*. elongation and myotube fusion) but eventually alters muscle development and causes muscle cell detachment. Actually, both factors are essential for the deployment of the myogenic transcriptional program, each of them controlling the activation of a subset of myogenic factors and markers, which collectively represent around half of the genes upregulated during HPM differentiation. A previous study showed that while P300 HAT activity was required for myogenesis in mouse embryos and embryoid bodies, CBP HAT activity appeared dispensable^48^. The difference between these results and ours might have several reasons. Notably, CBP and P300 HAT mutants behave as dominant negative^48^ and might thus cross-interfere with each other. Along that line, *myf5* expression, which is impaired in mouse mutants for P300 HAT activity^48^, was only affected in human primary myoblasts following the CBP and P300 double knockdown. Moreover, some HAT-independent functions for CBP have been described^49,50^. Finally, these discrepancies might reflect species or stage-specific differences. For example, it has been shown that *myogenin* plays distinct functions in mouse embryos and during post-natal life^51^. As human primary myoblasts were collected from adults, their differentiation probably recapitulates adult muscle growth and regeneration rather than embryonic myogenesis. Thus, in light of our results, we conclude that both CBP and P300 play an essential role in the activation of the adult muscle transcriptional program in human.

In addition to the regulation of muscle specific genes, our transcriptomic analyses highlight the role of CBP and P300 in the control of genes implicated in cell cycle and replication. In line with these results, it has been reported in other cell systems that CBP and P300 regulate replication^52^ and cell cycle progression^53^ and that C646 inhibits these processes^54^. Interestingly, our data suggest that they regulate cell cycle and replication in a complementary manner since we identified specific targets for CBP and P300 in these two processes.

Besides CBP and P300 specificity, the knockdown of both CBP and P300 revealed that they also play a redundant role for the regulation of certain genes. The two main categories of genes downregulated specifically upon CBP/P300 double knockdown correspond to muscle system and extracellular matrix organisation, which are also deregulated in single knockdown conditions. These two processes are thus targeted at multiple levels by CBP and P300. Moreover, the main hubs/genes upregulated after the double knockdown of CBP and P300 are implicated in apoptosis, suggesting that CBP and P300 play a major redundant role in cell protection and survival, which is consistent with the synthetic lethality recently described between CBP and P300 in cancer cells^32^. Of note, CBP/P300 double knockdown also caused an upregulation of HDAC1, which has been involved in inflammation and immune response^40^, but also in the repression of the myogenic program by modulating the transcriptional activity of MyoD^55,56^. Similarly, another histone deacetylase, SIRT3, which was shown to block myofibroblast differentiation^57^, and the methyltransferase G9a/EHMT2 (Euchromatic Histone Methyltransferase 2), which was found to inhibit adult myogenesis/regeneration^58–61^, were also upregulated. Hence, CBP and P300 knockdown promotes the expression of several epigenetic modifiers that might impair myogenesis.

Finally, we found that CBP and P300 downregulation in adult myoblasts leads to muscular dystrophy features. Indeed, one striking phenotype associated with CBP or P300 knockdown in differentiation-induced HPMs consisted in the massive detachment of the muscle fibers from the culture plate. Consistent with this, CBP and P300 were both required for the expression of several factors involved in cell adhesion, including components of the extracellular matrix, the sarcolemma and the cytoskeleton. In humans, muscular dystrophies include a heterogeneous group of genetic diseases leading to muscle degeneration and impaired function. Mutations of more than 30 genes give rise to various types of muscular dystrophies, which differ in severity, age of onset and muscle groups affected^62^. Mutations in the *Dystrophin* gene cause Duchenne and Becker muscular dystrophies, while mutations in *Sarcoglycan* are responsible for some forms of limb girdle muscular dystrophies^63^. Mutations in *Integrin α7* gene cause congenital myopathy^64^ and the most common form of congenital muscular dystrophy type 1 A (MDC1A) is associated with loss of functional Laminin α2^65^. We found that the knockdowns of CBP and/or P300 downregulated all afore mentioned genes and activated the expression of several genes associated with an inflammatory response, another characteristic feature of muscular dystrophies^66^. Interestingly, it has been shown that impaired muscle structure due to the absence of intracellular or extracellular structural components results in cell membrane instability, initiating a cascade of deleterious events such as apoptosis, necrosis and inflammation^62^. Strikingly, upon inhibition of CBP and P300 HAT activity in zebrafish, skeletal muscle development appeared normal but the first contractions triggered muscle cell detachment. This finding is similar to the phenotype reported for zebrafish dystrophic mutants and morphants^41–43^. Moreover, we recently showed that the enforced expression of CBP prevented muscle detachment in a zebrafish model of Duchenne muscular dystrophy^67^. Therefore CBP and P300 seem critical to sustain muscle integrity *in vivo*. On the opposite, previous studies have shown that deacetylase inhibitors (HDACi) can ameliorate dystrophic phenotypes by promoting myogenesis and regeneration in dystrophic muscles (for a review^68^). At the molecular level, *follistatin* (*Fst*) upregulation by HDACi was found to be responsible for boosting regeneration in dystrophic muscles^69,70^. Interestingly we observed that CBP or P300 knockdown decreases *follistatin* expression. Until now, a pharmacological treatment that cures muscle dystrophies still needs to be discovered. Epigenetic drugs such as HDACi, target the downstream effects of the genetic expression defects of *Dystrophin* or *-α-sarcoglycan*, but only slow down disease progression. It is tempting to speculate that the combination of HDACi with activators of CBP and P300 acetyltransferases activities^71^ could synergize to rescue muscle integrity and strength in muscular dystrophic diseases. These data highlight an unforeseen link between CBP/P300 activity and the emergence of dystrophic phenotypes and identify CBP and P300 as key mediators of adult muscle integrity, suggesting a new lead for intervention in muscular dystrophy.

## Materials and Methods

### Cell culture conditions

Primary human myoblasts were isolated from skeletal muscles of donors as previously described^72^. Purified myoblasts were plated in Petri dishes and cultured in growth medium containing Dulbecco’s Modified Eagle’s Medium (Gibco) supplemented with 20% foetal bovine serum (FBS) (GE Healthcare, PAA), 0.5% Ultroser G serum substitute (PALL life sciences) and 50 µg/ml Gentamicin (Thermo Scientific) at 37 °C in humidified atmosphere with 5% CO2. All experiments were carried out between Passage 4 (P4) and P8 to avoid cell senescence. Myogenic differentiation of confluent cells was induced by changing growth medium to DMEM containing 2% FBS and 50 µg/ml Gentamicin (differentiation medium).

### Ethics approval and consents

Human Primary myoblasts were collected in the Centre Hospitalier Universitaire Lapeyronie (Montpellier, France) from donors after obtention of their informed consent. All experiments were performed in accordance with the Declaration of Helsinki and approved by the ethical committee of the Hospital of Montpellier (France). Samples were approved for storage by the French “Ministère de l’Enseignement et de la Recherche” (N°DC-2008-594).

Zebrafish embryos were handled in a facility certified by the French Ministry of Agriculture (approval ID A-31-555-01) and in accordance with the guidelines from the European directive on the protection of animals used for scientific purposes (2010/63/UE), French Decret 2013–118. All experimental procedures were approved by the ethical committee of the “Ministère de l’Enseignement Superieur et de la Recherche et de l’Innovation” (approval number APAFiS#7124-2016100517263944v3).

### siRNA transfections

Myoblasts (0.25.10^6^ cells) were plated in 60 mm Petri dishes and cultured in growth medium for 48 h before being transfected with 5 nM siRNA using Lipofectamine RNAiMAX (Invitrogen) according to manufacturer’s instructions. Cells were harvested by scraping either at 24 h after transfection (T0) or at 24 h, 48 h and 72 h after induction of differentiation corresponding to T48, T72 and T96 from transfection time. Target sequences of siRNAs duplexes used are listed in supplementary methods.

### RNA extraction and transcriptome analysis

Total RNAs were extracted from cells using Aurum Total RNA Mini Kit (Bio-rad) according to manufacturer’s instructions and eluted in RNAse-free water. Biotinylated single strand cDNA targets were prepared, starting from 150 ng of total RNA, using the Ambion WT Expression Kit and the Affymetrix GeneChip® WT Terminal Labelling Kit according to Affymetrix recommendations. Following fragmentation and end-labelling, 3 µg of cDNAs were hybridized for 16 h at 45 °C on *GeneChip® Human Gene 2.0 ST arrays* (Affymetrix) interrogating over 400,000 RefSeq transcripts and ~ 11,000 LncRNAs represented by approximately 27 probes spread across the full length of the transcript. The chips were washed and stained in the GeneChip® Fluidics Station 450 (Affymetrix) and scanned with the GeneChip® Scanner 3000 7 G (Affymetrix) at a resolution of 0.7 µm. Raw data (.CEL Intensity files) were extracted from the scanned images using the Affymetrix GeneChip® Command Console (AGCC) version 3.2. CEL files were further processed with Affymetrix Expression Console software version 1.3.1 to calculate probe set signal intensities using Robust Multi-array Average (RMA) algorithms with default settings. The significance threshold for the dataset was calculated using a manual biostatistics analysis derived from Lenth’s pseudo standard error method^61^. Briefly, fold change values relative to siCTR were calculated at each time point and the values were ordered from the lowest (1 = no change) to the highest. Gene numbers were plotted on the x-axis and fold values were plotted on the y-axis. All values that departed from linearity were judged as significant. In order to determine a suitable cut off value for our datasets, we calculated average fold changes every 100 genes and the slopes of the corresponding curves. Fold changes were plotted on the x-axis and slopes on the y-axis. This showed that, depending on the siRNA treatment and the differentiation time point, the fold change values significantly departed from linearity from 1.5 to 1.75. Therefore, we chose 1.8 and used this value as our unique significance threshold for all our datasets. Of note, genes showing a significant change in expression between non-transfected (NT) cells and cells transfected with the control siRNA (siCTR) were not included in the present analysis to avoid effects due to the transfection itself and not the CBP and/or p300 knockdown.

### Heatmaps and gene networks construction

Lists of genes specifically regulated by CBP and/or P300 and by the double knock down of CBP and P300 were generated using a fold change of 1.8 relative to the corresponding time point transfected with the control siRNA (Table S1). These lists were subdivided in 4 cases on the basis of the influence of CBP and/or P300 knockdown, then classified on the basis on the gene expression levels (Hierarchical clustering) under the MeV environment^73^. Gene networks were created with Genomatix Pathway System (GePS) (www.genomatix.de) using a cut off of p-value < 10^−3^ for “pathway based networks” and “signal transduction networks” categories. The top-100 interacting genes were recovered and these interactions lists were then imported into Cytoscape version 3.6.0 (cytoscape.org)^74^ to visualize the networks and the hubs using the prefuse force directed layout. Node sizes and labels were based on the degree of connectivity of the nodes. Hence, larger nodes represent network hubs.

### Reverse transcription and real-time PCR

1 µg total RNA was converted into cDNA using iScript cDNA Synthesis Kit (Bio-rad) with Oligo(dT) and random hexamer primers for 30 min at 42 °C according to manufacturer’s instructions. cDNA were then diluted 20-fold and quantified by qPCR using SsoFast Evagreen Supermix (Bio-rad) and sequence specific primers. Data were acquired on CFX96 Real-Time System (Bio-rad). Samples were analyzed in triplicates and the expression level was calculated relative to *GAPDH* for human cDNA and to *EF1α* for zebrafish samples. Sequences of primer pairs used in gene expression analysis are listed in supplementary methods.

### Protein isolation and Western-blot analyses

Cells were lysed in Protein extraction buffer (50 mM Tris pH 8, 150 mM NaCl, 0.5% NP40 and 1 mM EGTA) and protein concentrations were determined using the Bio-Rad DC Protein Assay reagents package (Bio-rad). A total of 40 µg of total proteins were separated by SDS-PAGE and transferred to Nitrocellulose membranes, blocked 1 h at room temperature (10% milk, 0.1% Tween-20 TBS) and probed at 4 °C overnight (2% milk, 0.1% Tween-20 TBS) with the following primary antibodies: anti-CBP (sc-369, Santa Cruz Biotechnology); anti-P300 (sc-585, Santa Cruz Biotechnology); anti-Myf5 (sc-302, Santa Cruz Biotechnology); anti-Myogenin (sc-576, Santa Cruz Biotechnology); anti-Mef2C (sc-13266, Santa Cruz Biotechnology); anti-Vinculin (sc-5573, Santa Cruz Biotechnology) and anti-MHC (MF20, Developmental Studies Hybridoma Bank). After washings, membranes were incubated with horseradish peroxidase-conjugated secondary antibodies for 1 h. Detection was performed using Lumi-light^plus^ Western Blotting substrate (Roche) according to manufacturer’s instruction. Original scans of all western blots shown in this study can be found in Supplementary Fig. S7.

### Zebrafish lines and embryo injections

Zebrafish transgenic line Tg*(smyhc1:EGFP)*^*i104*^^75^ was maintained on wild-type background. Staging and husbandry were as described^76^.

Embryos were treated with 2 μM or 3.5 μM of C646 CBP/P300 inhibitor (Calbiochem) or with DMSO for different periods of time as indicated. Immunohistochemistry experiments were performed as followed. Embryos were fixed with 4% paraformaldehyde (PFA)/PBS overnight at 4 °C, then washed twice with Phosphate Buffered Saline/1% Triton X-100 (PBST), permeabilized with PBST/0.5% Trypsin for 30 sec (>20 hpf embryos) or for 1 min (>48 hpf embryos) and washed twice again with PBST. After blocking with PBST/10% Foetal Calf Serum (FCS)/1% bovine serum albumin (BSA) (hereafter termed ‘blocking solution’) for at least 1 h, embryos were incubated with antibodies directed against either GFP (Torrey Pine, Biolabs), or acetyl-H3K27 (Abcam), or acetyl-p53 (Cell Signalling Technology) or cleaved Caspase-3 (Asp175) (Cell Signaling Technology) in blocking solution overnight at 4 °C followed by washing five times with PBST. Embryos were then incubated with the appropriate Alexa Fluor-conjugated secondary antibodies (Molecular Probes) for at least 2 h at room temperature and washed three times. Nuclei were then stained with TO-PRO3 (Molecular Probes) and washed twice with PBST. Embryos were dissected, flat-mounted in glycerol and images were recorded on a confocal microscope (TCS SP8, Leica Microsystems) with an L 25 × /0.95 W FLUOTAR VISIR objective (zoom X1.25) using the scanner resonant mode.

## Electronic supplementary material

Supplementary Information

Table S1

Table S2

Table S3

Table S4

## Data Availability

The microarray data from this study were submitted and approved by the National Center for Biotechnology Information (NCBI) Gene Expression Omnibus (GEO) database. They are available under the accession number GSE94006.
