# Supplementary material for: CBP and P300 regulate distinct gene networks required for human primary myoblast differentiation and muscle integrity
Source: Sci Rep. 2018 Aug 22;8:12629. doi: 10.1038/s41598-018-31102-4 (PMC6105712; doi:10.1038/s41598-018-31102-4)
Supplement: Supplementary file 1 — Supplementary Information [file 41598_2018_31102_MOESM1_ESM.pdf]

Supplementary Information

CBP and p300 regulate distinct gene networks required for human primary myoblast differentiation and muscle integrity

Lucas Fauquier[1#], Karim Azzag[1,2#], Marco Antonio Mendoza Parra[3], Aurélie Quillien[1], Manon Boulet[1,4], Sarah Diouf[1,4], Gilles Carnac[5], Lucas Waltzer[1,4], Hinrich Gronemeyer[3], Laurence Vandel[1,4*]

Supplementary Fig. S1 to S8

Supplementary Table Legends

Supplementary Methods

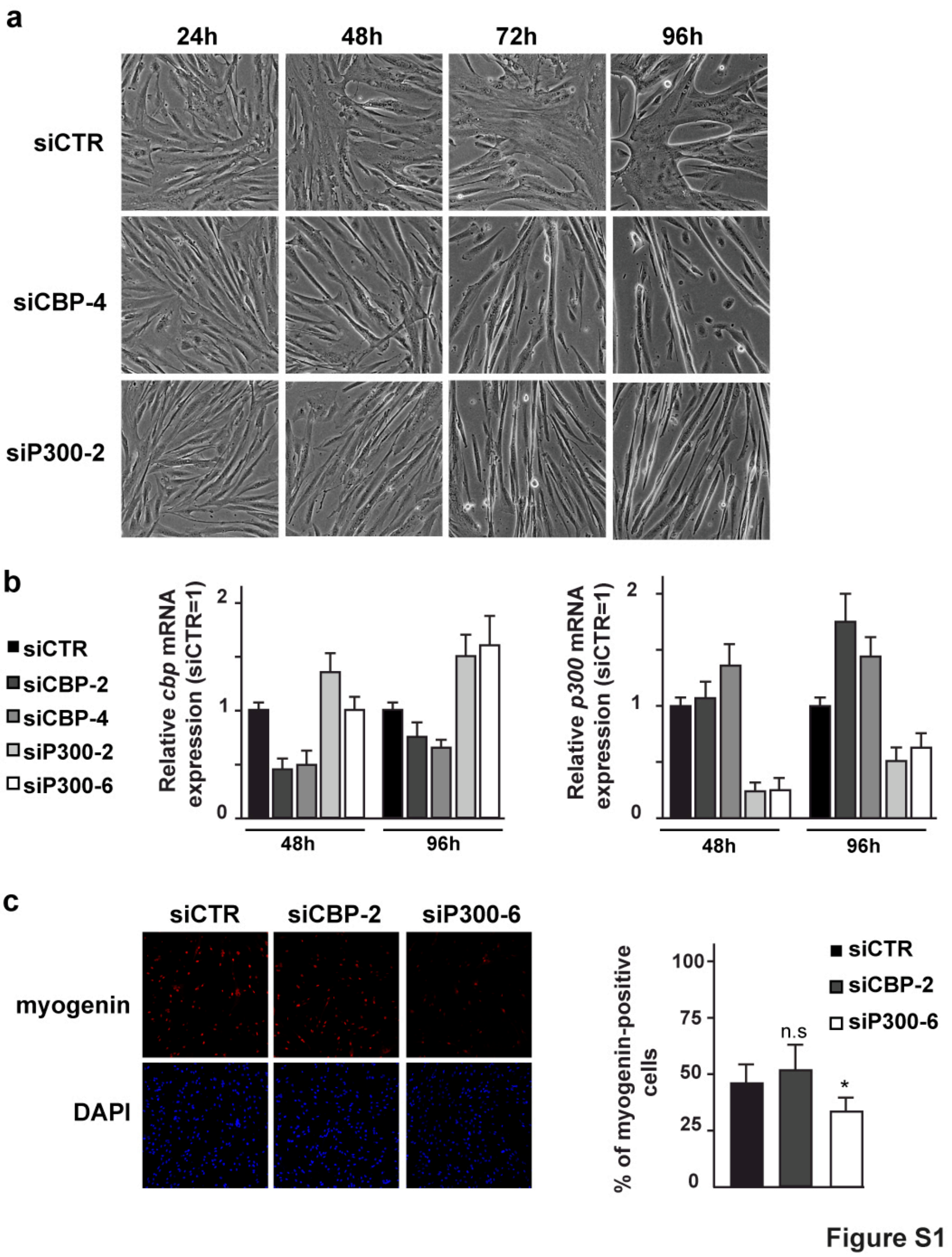

Fig. S1 : Independent siRNAs directed against CBP and P300 give rise to similar cell phenotypes as in Fig. 2.
(**a**) Phase contrast photographs at different time points of differentiation of Human Primary Myoblasts (HPM) transfected with the indicated siRNAs. (**b**) RT-qPCR of CBP and P300 expression after transfection with the indicated siRNAs at 48h and 96h of transfection to validate the efficiency of the siRNAs. Expression was relative to GAPDH transcript and set at 1 in the siRNA control (siCTR) condition at all time points. (**c**) Analysis of MYOGENIN expression by immunofluorescence in HPM transfected with the indicated siRNAs at 48h after transfection (left panel) with the corresponding quantifications (right panel). Error bars represent S.D. Statistical significance are relative to siCTR and were calculated with an unpaired t-test; ns: not significant; * $p<0.05$.
A minimum of 5 fields from 2 independent experiments were analysed per condition.

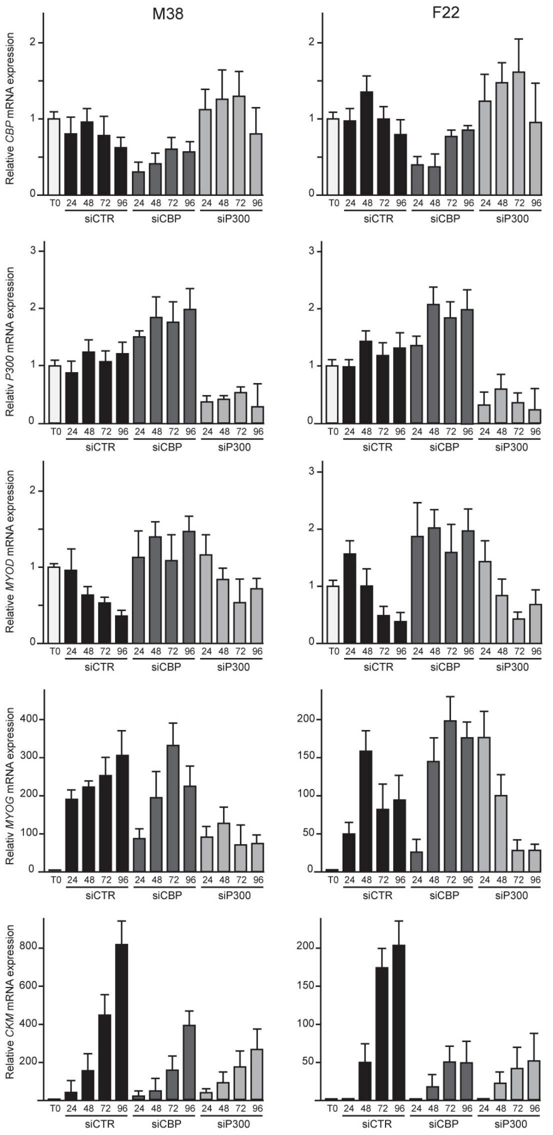

Figure S2

Fig. S2 : Donors from different age and sex display similar expression changes of selected gene targets after CBP and P300 knockdowns as in fig. 2. RT-qPCR were performed with RNA extracted from 2 independent patients of different age and sex at different time points of differentiation after siRNA transfection as indicated. M38 was a 38-year old male distinct from the M39 male used throughout the study and F22 was a 22-year old female. A similar effect of CBP or P300 knockdown on MYOD, MYOGENIN and CKM expression was observed in M39, M38 and F22 donors but with different response ranges. As siCBP was less efficient in M38 and F22 donors than in M39, donor M39 was chosen for gene network analyses and all subsequent work presented in this study. Error bars represent S.D from 3 independent experiments.

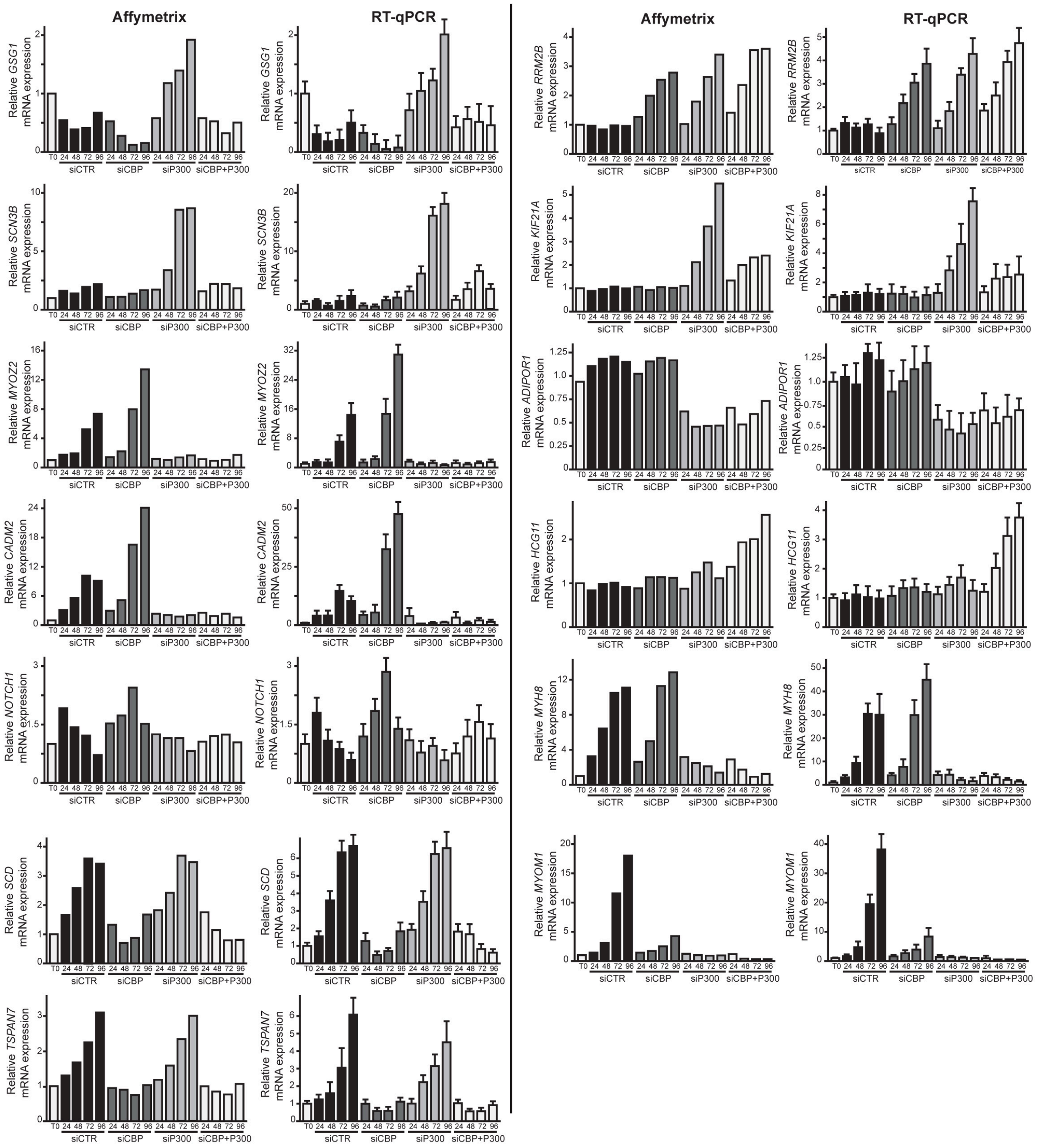

Figure S3

Fig. S3 : Validation of Affymetrix data by RT-qPCR on selected target genes. Affymetrix expression data of the indicated genes (left panels) after transfection of the indicated siRNAs and at different time points of differentiation were compared to data derived from RT-qPCR experiments for the same genes (right panels). Note the similar expression patterns between Affymetrix and RT-qPCR data but with a general higher expression in RT-qPCR experiments. Error bars represent S.D from 3 independent experiments.

a **Number of genes down-regulated after transfection with siRNAs against CBP, P300 and CBP+P300 at different time points**

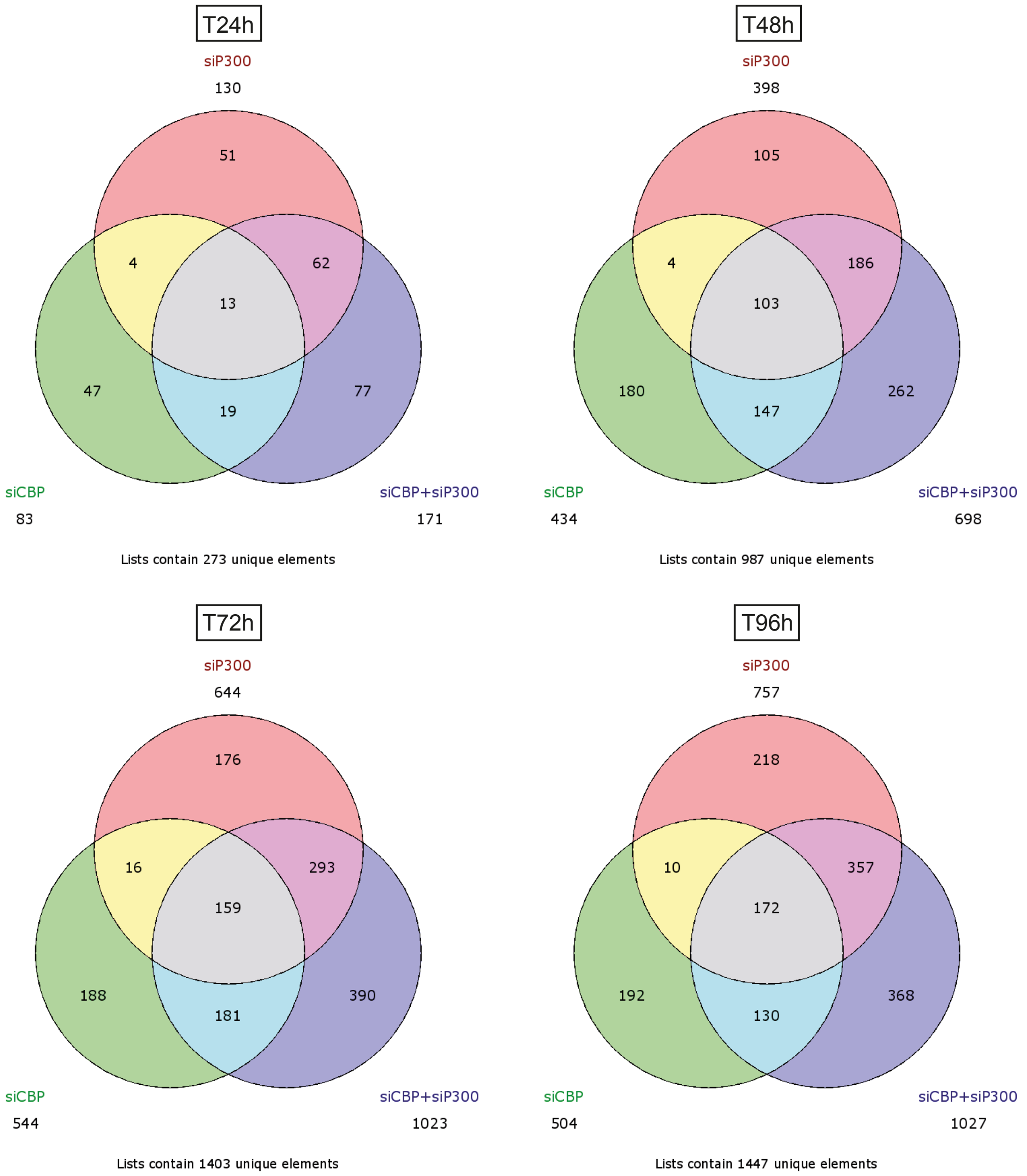

Figure S4a

b **Number of genes up-regulated after transfection with siRNAs against CBP, P300 and CBP+P300 at different time points**

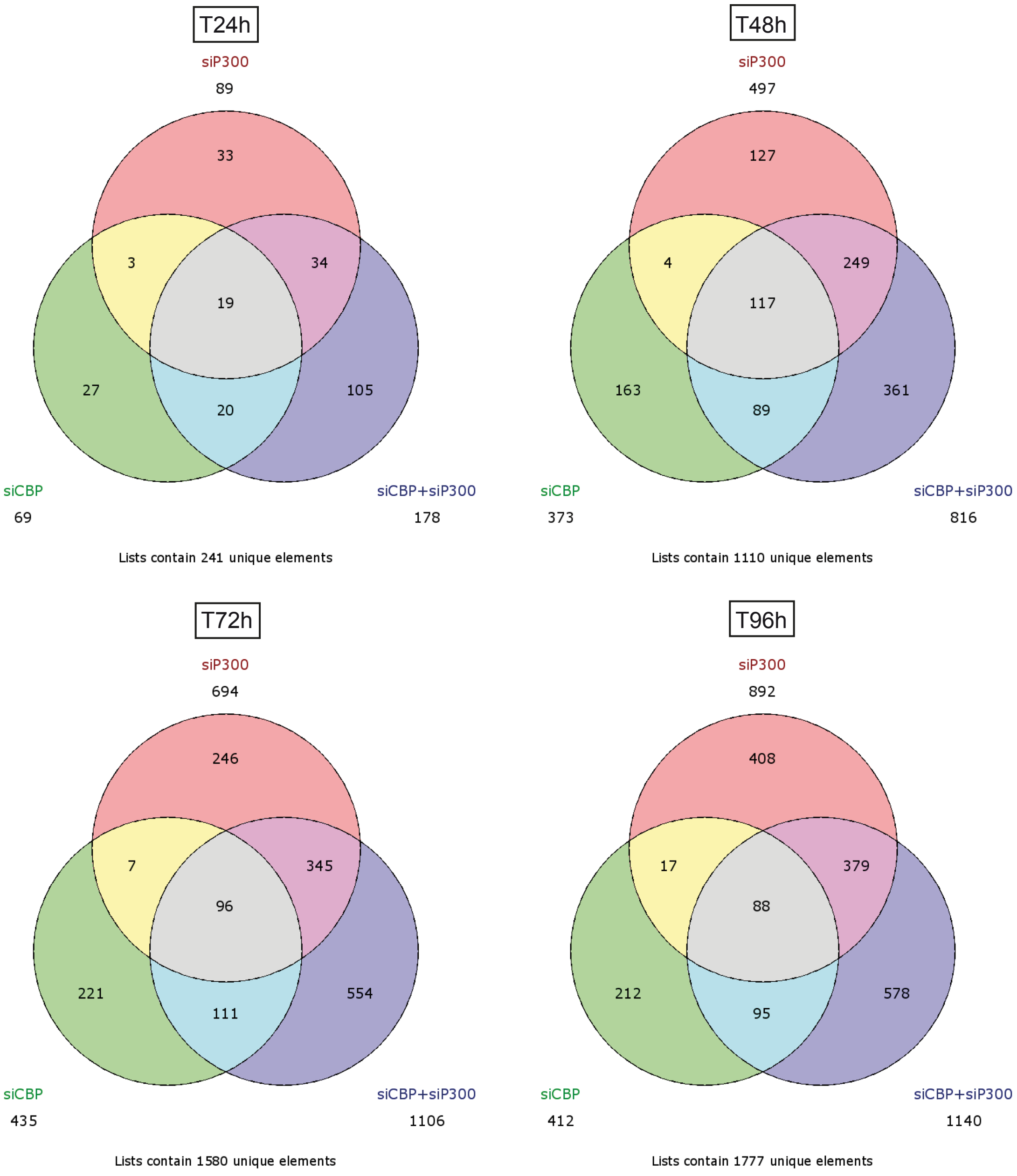

Figure S4b

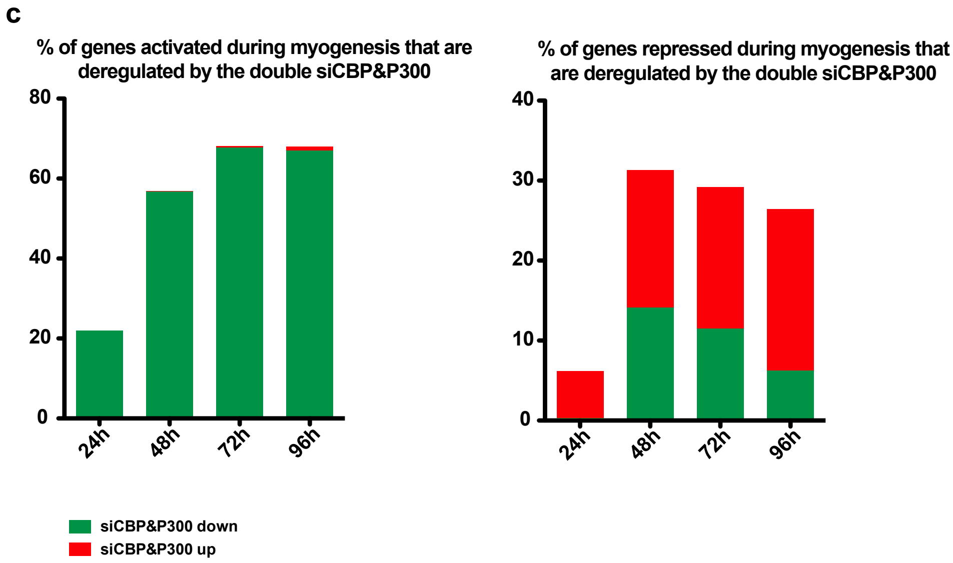

Figure S4c

Fig. S4 : Knockdowns of CBP or P300 mostly downregulate (a) or upregulate (b) distinct gene populations during differentiation while the double knockdown identifies genes for which CBP and p300 are redundant. Venn diagrams were created from the lists of genes from Table S1 with the Venn diagram Generator Pangloss wisdom software. (c) Proportion of genes activated (left panel) or repressed (right panel) during HPM differentiation that are affected by the double siCBP&P300.

a

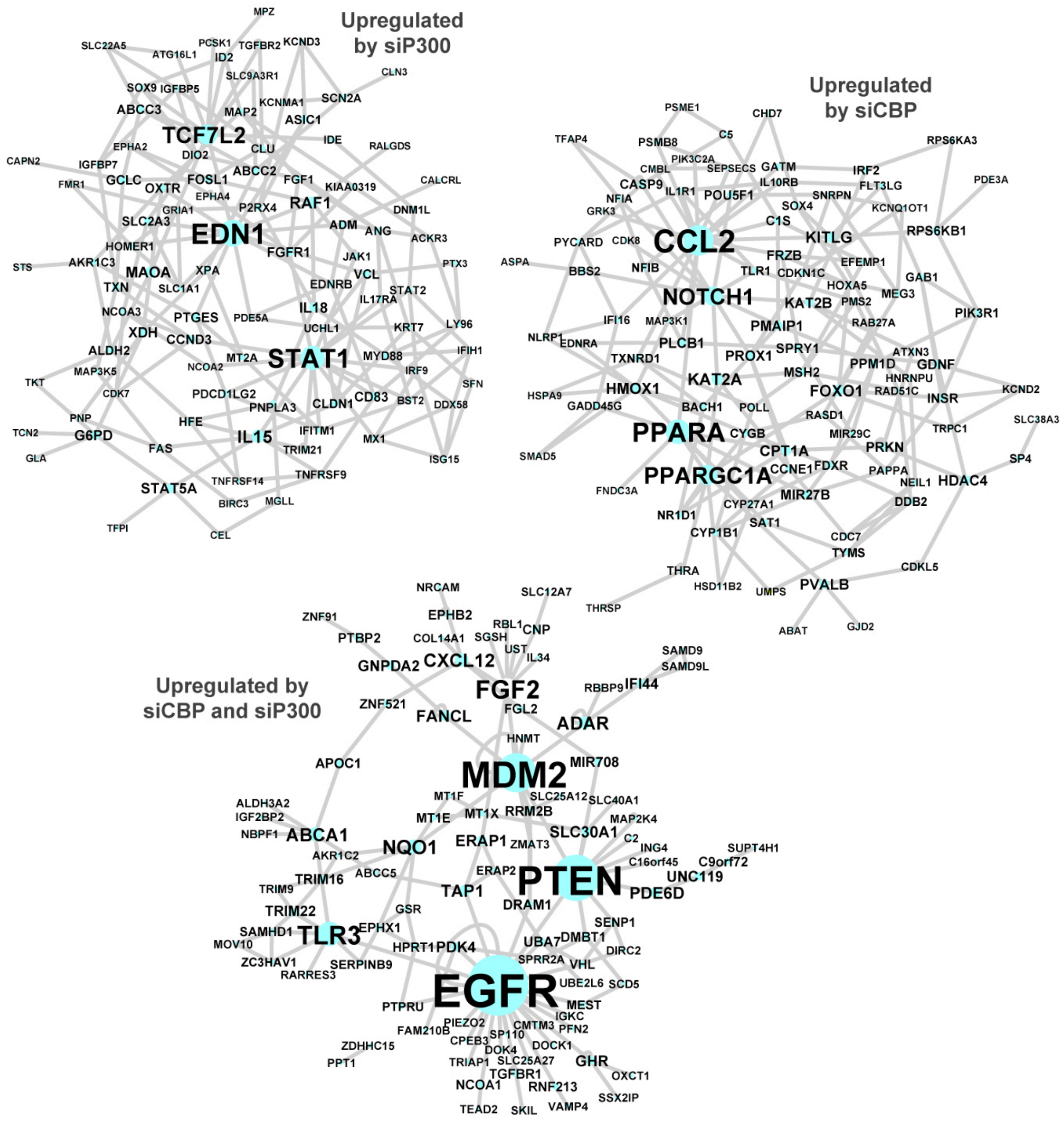

Upregulated by siP300
MPZ
SLC22A5
ATG16L1
PCSK1
ID2
TGFBR2
KCND3
SLC9A3R1
CLN3
SOX9
IGFBP5
KCNMA1
SCN2A
ABCC3
MAP2
ASIC1
TCF7L2
IDE
RALGDS
EPHA2
CLU
DIO2
CAPN2
IGFBP7
ABCC2
FMR1
GCLC
FOSL1
FGF1
CALCRL
OXTR
KIAA0319
EPHA4
GRIA1
P2RX4
RAF1
DNM1L
SLC2A3
ADM
EDN1
ANG
ACKR3
HOMER1
FGFR1
STS
AKR1C3
JAK1
PTX3
MAOA
XPA
VCL
TXN
SLC1A1
EDNRB
STAT2
IL17RA
NCOA3
IL18
PTGES
PDE5A
LY96
XDH
UCHL1
KRT7
CCND3
ALDH2
MT2A
STAT1
MYD88
IFIH1
NCOA2
MAP3K5
IRF9
TKT
CDK7
SFN
PDCD1LG2
CD83
BST2
DDX58
PNPLA3
CLDN1
PNP
HFE
TCN2
G6PD
IFITM1
IL15
MX1
TRIM21
GLA
FAS
ISG15
TNFRSF9
TNFRSF14
STAT5A
BIRC3
MGLL
TFPI
CEL
Upregulated by siCBP
PSME1
CHD7
C5
TFAP4
PSMB8
RPS6KA3
PIK3C2A
CMBL
SEPSECS
GATM
IRF2
CASP9
IL10RB
PDE3A
FLT3LG
NFIA
IL1R1
POU5F1
SNRPN
GRK3
SOX4
KCNQ1OT1
C1S
PYCARD
CDK8
CCL2
KITLG
RPS6KB1
ASPA
NFIB
BBS2
FRZB
EFEMP1
TLR1
CDKN1C
GAB1
HOXA5
NOTCH1
KAT2B
PMS2
MEG3
IFI16
MAP3K1
PIK3R1
PMAIP1
RAB27A
NLRP1
EDNRA
PLCB1
SPRY1
ATXN3
TXNRD1
PROX1
PPM1D
GDNF
KAT2A
MSH2
HNRNPU
HSPA9
HMOX1
FOXO1
RAD51C
KCND2
GADD45G
BACH1
POLL
INSR
RASD1
SLC38A3
PPARA
CYGB
TRPC1
SMAD5
MIR29C
CPT1A
PRKN
SP4
PPARGC1A
CCNE1
FDXR
PAPPA
FNDC3A
NEIL1
HDAC4
CYP27A1
MIR27B
DDB2
NR1D1
SAT1
CYP1B1
CDC7
TYMS
THRA
CDKL5
HSD11B2
UMPS
PVALB
THRSP
ABAT
GJD2
NRCAM
SLC12A7
EPHB2
ZNF91
RBL1
CNP
PTBP2
COL14A1
SGSH
UST
IL34
CXCL12
GNPDA2
SAMD9
SAMD9L
Upregulated by siCBP and siP300
FGF2
RBBP9
IFI44
ZNF521
FGL2
FANCL
ADAR
HNMT
APOC1
MDM2
MIR708
MT1F
SLC25A12
SLC40A1
ALDH3A2
RRM2B
IGF2BP2
MT1E
MT1X
MAP2K4
NBPF1
ABCA1
SLC30A1
ERAP1
C2
ING4
ZMAT3
NQO1
SUPT4H1
AKR1C2
C16orf45
C9orf72
ABCC5
ERAP2
UNC119
TRIM9
TRIM16
PTEN
TAP1
PDE6D
TRIM22
GSR
DRAM1
SENP1
SAMHD1
TLR3
EPHX1
MOV10
UBA7
DMBT1
HPRT1
PDK4
DIRC2
ZC3HAV1
SERPINB9
SPRR2A
VHL
RARRES3
EGFR
UBE2L6
SCD5
PTPRU
MEST
IGKC
PIEZO2
CMTM3
PFN2
FAM210B
SP110
CPEB3
ZDHHC15
DOK4
DOCK1
PPT1
TRIAP1
SLC25A27
GHR
TGFBR1
OXCT1
NCOA1
RNF213
SSX2IP
TEAD2
SKIL
VAMP4

Figure S5a

b

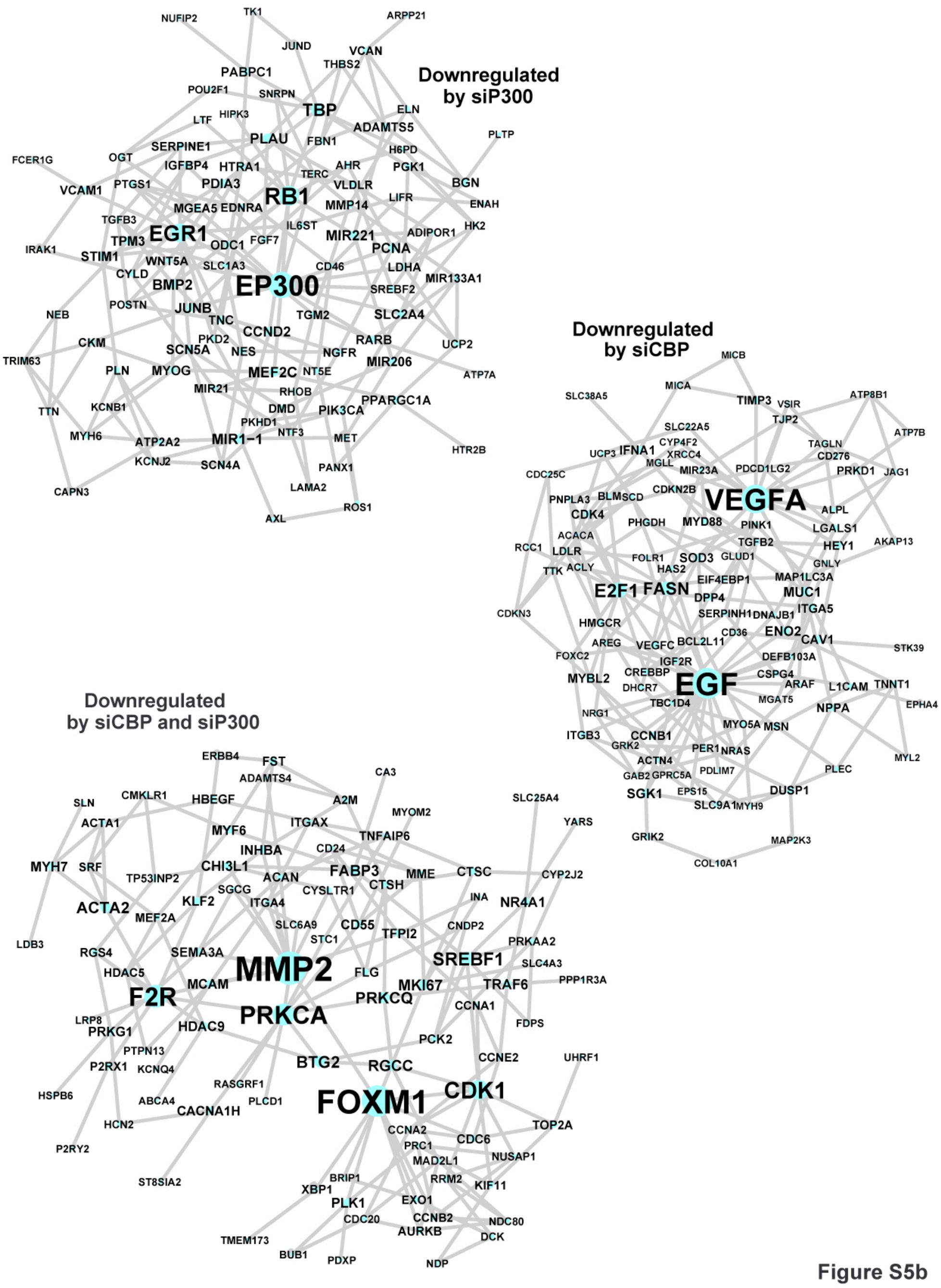

Figure S5b

Fig. S5 : Common and divergent gene regulatory networks that are upregulated (a) or downregulated (b) after CBP or P300 knockdown. Gene networks were created with Genomatix Pathway System (GePS) (www.genomatix.de) from network interaction lists. These interactions lists were then imported into Cytoscape (cytoscape.org) to visualize the networks and the hubs using the prefuse force directed layout. Node sizes and labels were based on the degree of connectivity of the nodes. Hence, larger nodes represent network hubs.

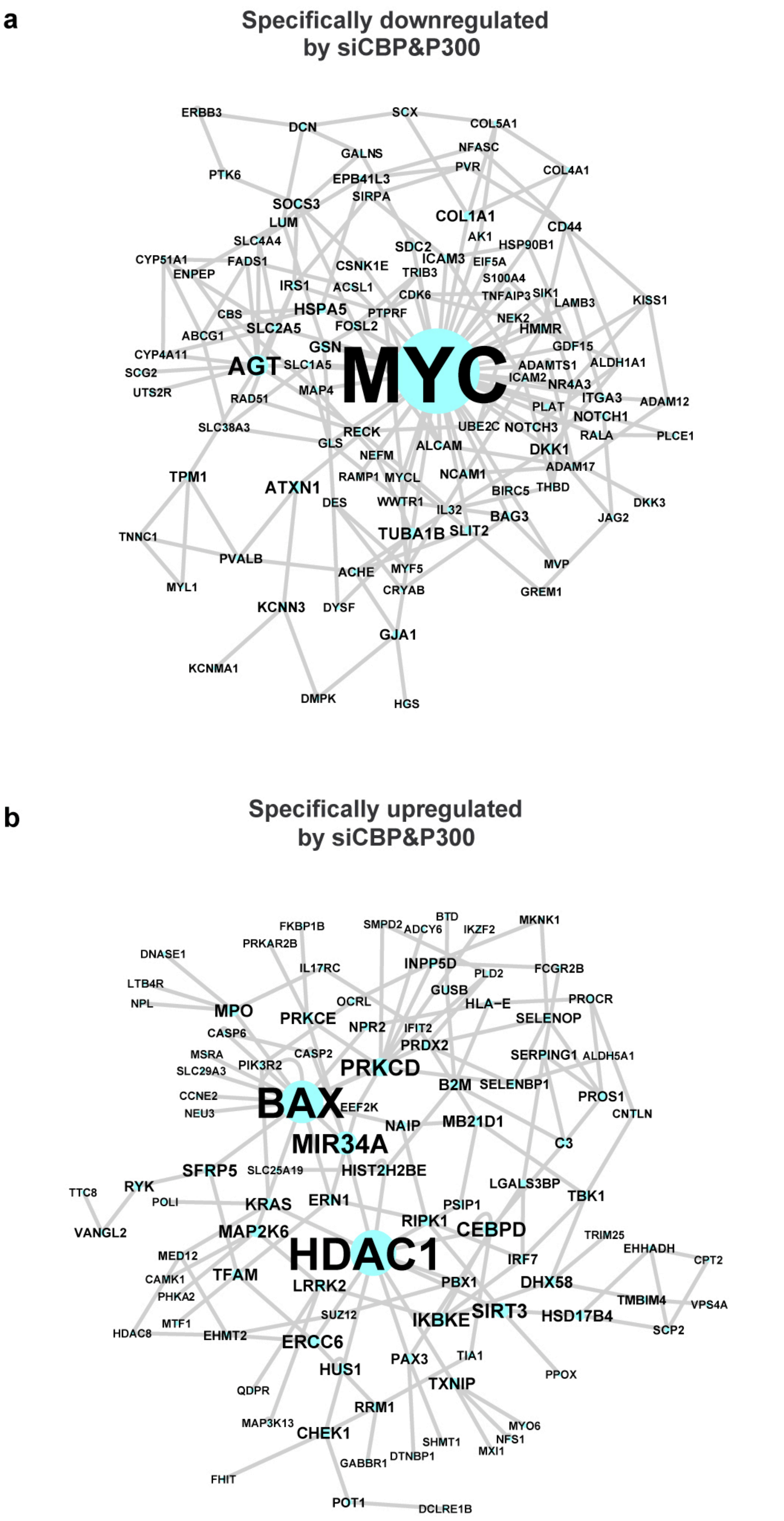

Figure S6

Fig. S6: Specific gene regulatory network downregulated (**a**) or upregulated (**b**) after CBP & P300 knockdown. Gene network was created as in Suplementary Fig S5.

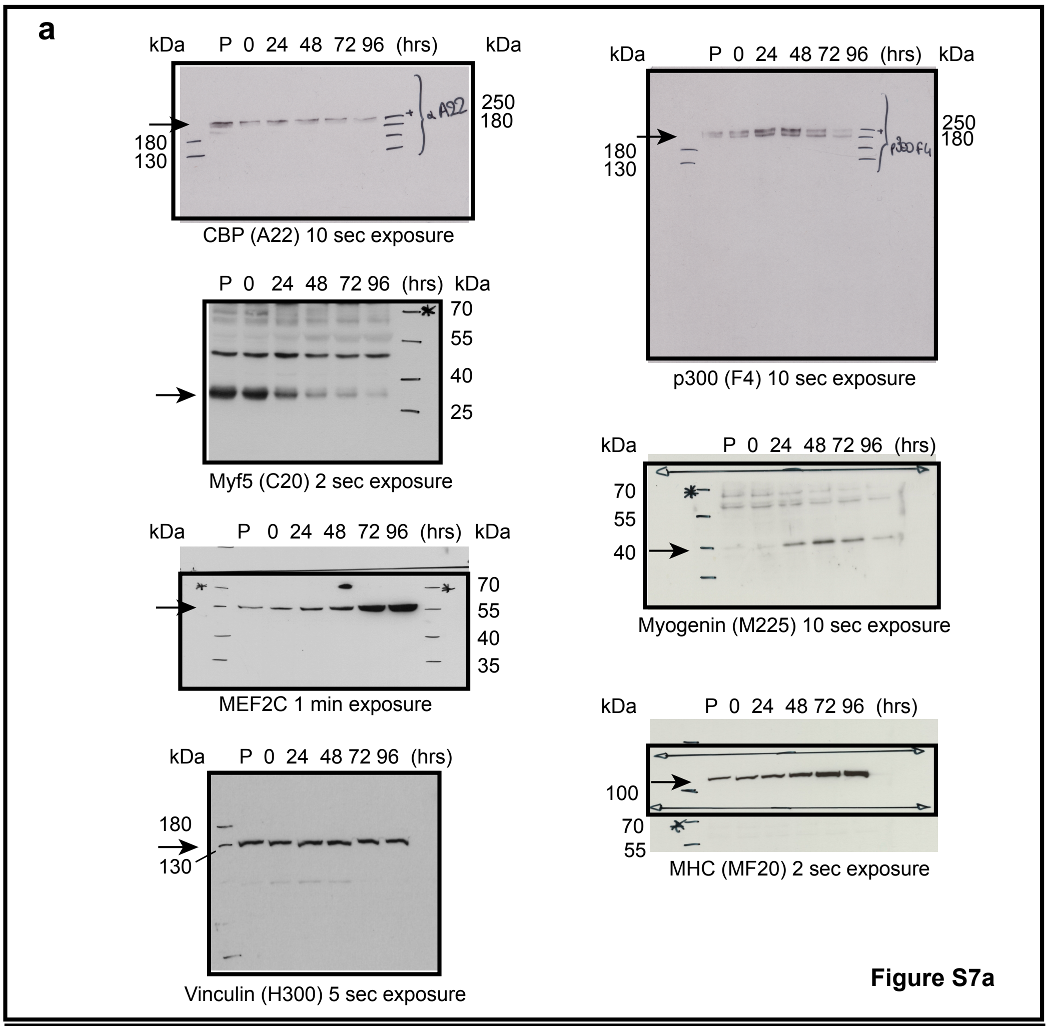

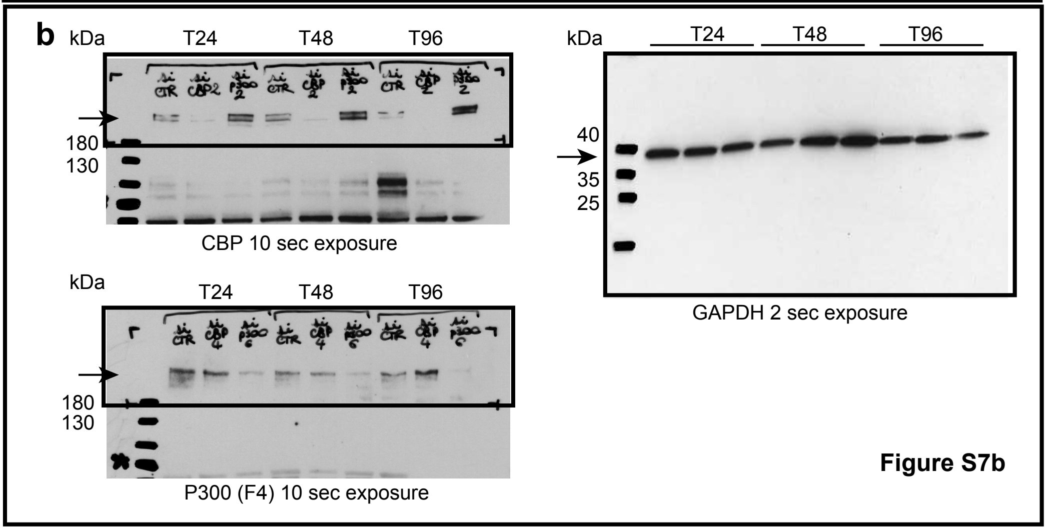

Fig. S7 : Original Scans of western blots as shown in Figures 1 (a) and 2 (b). Zones probed with the indicated antibodies are boxed. Arrows show the size of the protein of interest.

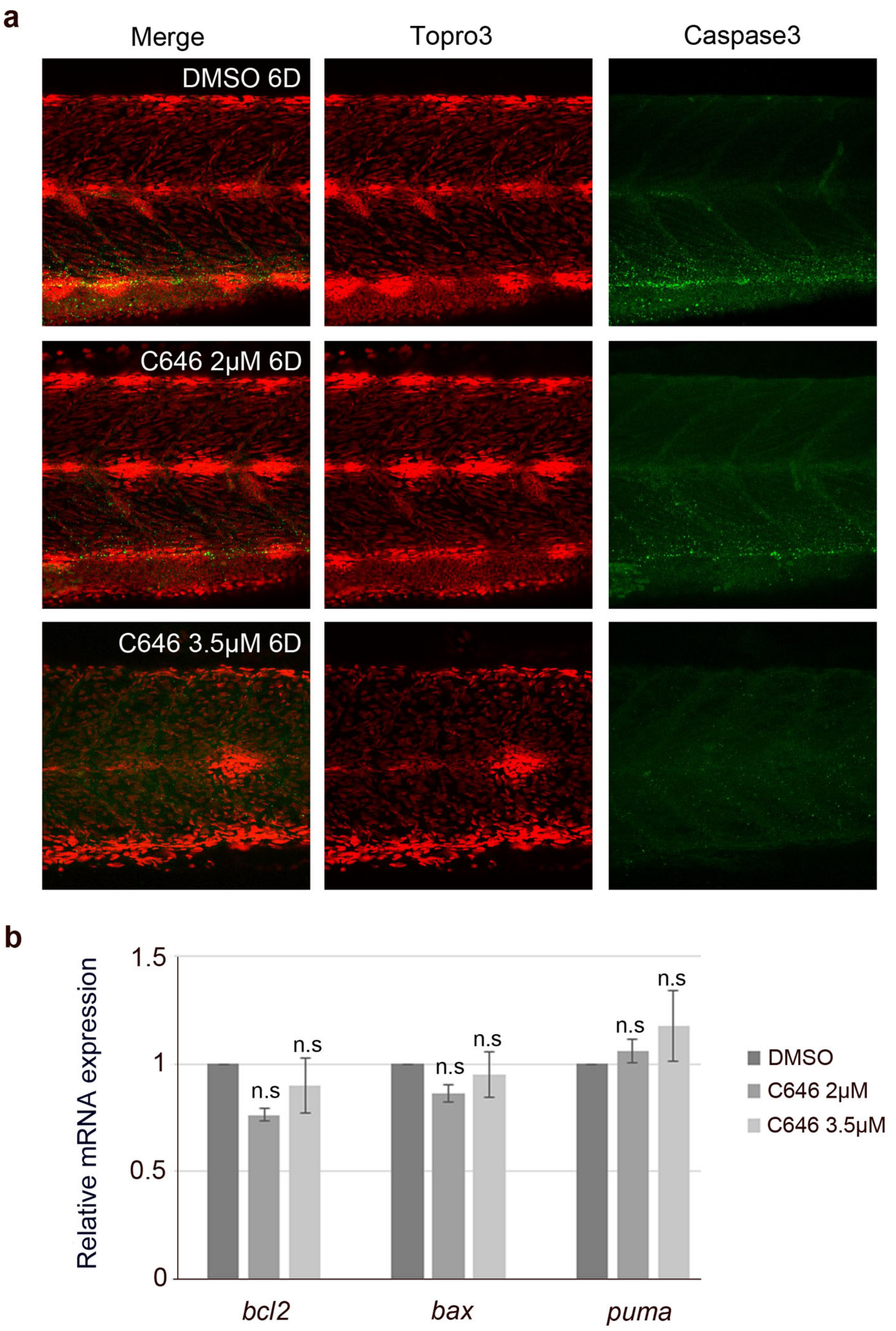

Figure S8

Fig. S8: P300/CBP histone acetyltransferase activity inhibition does not induce apoptosis in muscle cells when fibers detach in zebrafish
(**a**) Confocal projections of embryos treated with either DMSO (vehicle), 2 μM or 3.5 μM C646 at 10 hpf for 24 h, fixed and immunostained against caspase 3 and imaged at 6 dpf (6D). Topro3 was used to stain the nuclei.(**b**) Relative mRNA expressions of *bcl2*, *bax* and *puma* were determined by RT-qPCR in 3 dpf embryos treated with either DMSO, or 2 μM or 3.5 μM C646 from 10 hpf for 24 h. Error bars represent S.D of 3 independent experiments. Statistical significance is relative to control and was calculated with an unpaired t-test. ns: not significant. A minimum of 12 embryos were analysed per condition.

Supplementary Table Legends

Supplementary Table S1: (**a**) Expression data at different time points of human primary differentiation of genes deregulated by siCBP or siP300 by a fold change of 1.8 compared to siCTR. Color code of the gene symbols is the following: Genes specifically down- and upregulated by siP300 are in grey and orange, respectively; genes specifically down- and upregulated by siCBP are in brown and green, respectively and genes that are regulated in an opposite way by CBP and P300 are in burgundy and yellow. Genes specifically up or downregulated by the double siCBP&P300 are in dark grey. These gene lists were used for building heatmaps (Fig. 3a-d). (**b**) Lists and number of genes that are activated or repressed during differentiation in siRNA control conditions.

Supplementary Table S2 : Lists and number of genes specifically or commonly up- or downregulated by CBP and/or P300 at all time points of differentiation**.** These lists were used to create the Venn diagrams in Supplementary Fig. S4.

Supplementary Table S3 : Lists of genes that are upregulated (in red) or downregulated (in green) by siCBP, siP300 or by the double knockdown siCBP&P300, as indicated. Gene Ontology (GO) analyses of each class of genes as indicated using Genomatix software (www.genomatix.de).

Supplementary Table S4 : Comparison of the genes deregulated by CBP and/or P300 in this study with annotated genes associated with active enhancers described in [31]. (**a**) Gene ontology (GO) analysis of the genes down- or upregulated by CBP or

P300 single knockdown or by the double knockdown CBP&P300 described as top-scoring most representative GOs in Table 1, that are associated with active enhancers identified in [31]. GOs associated with downregulated genes by siCBP, siP300 or siCBP&P300 are in green, while GOs associated with upregulated genes are in red. Numbers refer to p-values (“n.s.”: not significant / p-value $\geq 10^{-3}$). (**b,c,d**) Lists of genes that are upregulated or downregulated by siCBP, siP300 or by the double knockdown siCBP&P300 and that are associated with active enhancers, as indicated. (**e,f,g**) Full Gene Ontology (GO) analyses of each list of genes (b,c,d) using Genomatix software (www.genomatix.de).

Supplementary methods: (**a**) siRNA target sequences used in this study. (**b**) primers pairs used in real time PCR.

SUPPLEMENTARY METHODS

a. SiRNA transfections

The following siRNA target sequences were used in this study:

NAME	SEQUENCE
siCTR	CATGTCATGTGTCACATCT
siCBP-2	AACAGGCAGCCAGCACCTCTG
siCBP-4	GATGCTGCTTCCAAACATA
siP300-2	TTGGACTACCCTATCAAGTAA
siP300-6	GGGAATGAATGTAACAAAT

Table 1: siRNA target sequences

b. Real-time PCR

The following primers were used in this study:

GENE	FORWARD PRIMER 5'->3'	REVERSE PRIMER 5'->3'
CBP	TGCCAAGTTGCCCATTGTG	TTGTTGGTTTCGCTTGTCACT
P300	TTCAAACGCCGAGTCTTCTT	GTTGAGCTGCTGTTGGCATA
MYOG	CAGTGCCATCCAGTACATCG	AGGTTGTGGGCATCTGTAGG
MYO	TCCAGCCCGCGCTCCAACTGC	TCGACACCGCCGCACTCTTCC
MYF5	AATTTGGGGACGAGTTTGTG	CATGGTGGTGGACTTCCTCT
MEF2C	TTTGTGCCATCTGCCGTGATGT	ATGTGGCTTGGATTGCTCCACA
GSG1	TTCAAGGAAAACCCGAACTG	GGTGGTAGCTGGTCAAAGGA
SCN3B	CCACACACCCAGACTTCCTT	CAGCACATTCCTGCTTTGAA
MYOZ2	TCCAGACAACATTGCTCCAG	CTTGTTCCCAGGGAGATTGA
CADM2	TTGTGATGTCTGACGCATGA	CATGATGTTGCACTCAACCA
TSPAN7	CTGGGGCAAACTTACTCTGG	CAAACAGGCCAAAGACAACA
SCD	CCACCGCTCTTACAAAGCTC	GGTCACGAGCCCATTCATAG
MYH7	CACGCAATATCAAGGGGAAGCC	GGTGCTGAGCAGCTTGAGGGAA
MYH8	GCAAAACGGAAGGAGCTAGA	TGCATCAGCCAAGCTATCTG
ADIPOR1	CTTGACCATGCTCAGACCAA	GGTGTGAAAGAGCCAGGAGA
HCG11	CGGTTGGTCTCCGTACTACC	CGTAAACGGCCTGTGAATCT
GAPDH	TGCACCACCAACTGCTTAGC	GGCATGGACTGTGGTCATGAG
KIF21	GCAGATGGAAGAAGCAAAGG	GATGCCCATTGACAGGAAGT
NOTCH1	GAAAATGTGTTCTCGGAGTGTG	TGGAGGGACCAAGAACTTGTAT
RRM2B	GGATCTCCCTCACTGGAACA	GACTAAAGCGCTCCACCAAA
CKM	ATGCCATTCGGTAACACCCACA	TTGTCCCGCAGCTTCTTGTAGA
MYOT	CAAGCTCAGGATGACAGTGGT	TCATTCACATCTCCCCTTGAG
MSTN	ACTTCGTCTGGAAACAGCTCCT	AAAGAGCCATCGCTGCTGTCAT

Table 2 : sequences of primer pairs
